# Synthesis, Biological and In Silico Studies of Griseofulvin and Usnic Acid Sulfonamide Derivatives as Fungal, Bacterial and Human Carbonic Anhydrase Inhibitors

**DOI:** 10.3390/ijms24032802

**Published:** 2023-02-01

**Authors:** Andrea Angeli, Anthi Petrou, Victor Kartsev, Boris Lichitsky, Andrey Komogortsev, Clemente Capasso, Athina Geronikaki, Claudiu T. Supuran

**Affiliations:** 1 NeuroFarba Department, Sezione di ScienzeFarmaceutiche, Università degli Studi di Firenze, Via Ugo Schiff 6, 50019 Sesto Fiorentino, Italy; 2 Istituto di Bioscienze e Biorisorse, CNR (National Research Council), Via Pietro Castellino 111, 80131 Napoli, Italy; 3 Department of Pharmacy, School of Health, Aristotle University of Thessaloniki, 54124 Thessaloniki, Greece; 4 InterBioScreen, Chernogolovka 142432, Russia; 5 Zelinsky Institute of Organic Chemistry, Leninsky Prospect, Moscow 119991, Russia

**Keywords:** griseofulvin derivatives, usnic acid derivatives, carbonic anhydrase inhibitors, stopped-flow CO_2_ hydrase assay, in silico studies

## Abstract

Carbonic anhydrases (CAs, EC 4.2.1.1) catalyze the essential reaction of CO_2_ hydration in all living organisms, being actively involved in the regulation of a plethora of patho-/physiological conditions. A series of griseofulvin and usnic acid sulfonamides were synthesized and tested as possible CA inhibitors. Since β- and γ- classes are expressed in microorganisms in addition to the α- class, showing substantial structural differences to the human isoforms they are also interesting as new antiinfective targets with a different mechanism of action for fighting the emerging problem of extensive drug resistance afflicting most countries worldwide. Griseofulvin and usnic acid sulfonamides were synthesized using methods of organic chemistry. Their inhibitory activity, assessed against the cytosolic human isoforms hCA I and hCA II, the transmembrane hCA IX as well as β- and γ-CAs from different bacterial and fungal strains, was evaluated by a stopped-flow CO_2_ hydrase assay. Several of the investigated derivatives showed interesting inhibition activity towards the cytosolic associate isoforms hCA I and hCA II, as well as the three γ-CAs and *Malassezia globosa* (MgCA) enzyme. Six compounds (**1b**–**1d**, **1h, 1i** and **1j**) were more potent than AAZ against hCA I while five (**1d**, **1h**, **1i**, **1j** and **4a**) showed better activity than AAZ against the hCA II isoform. Moreover, all compounds appeared to be very potent against MgCA with a Ki lower than that of the reference drug. Furthermore, computational procedures were used to investigate the binding mode of this class of compounds within the active site of human CAs.

## 1. Introduction

Carbonic anhydrases (CAs, EC 4.2.1.1) are a group of metalloenzymes implicated in pH buffering of extra- and intracellular spaces by catalyzing the reversible hydration of carbon dioxide (a cellular waste product) to bicarbonate and a proton [1,2,3,4]. This family of enzymes to date, is divided in eight independent gene families (i.e., α, β, γ, δ, ζ, η, θ and ι-classes) Among the 15 known humans (h) CA isoforms belonging to class α, the cytosolic isoforms hCA I and hCA II are omnipresent in the body and represent the targets for anticonvulsant, diuretic, and anti-glaucoma drugs. On the other hand, the transmembrane isoforms hCA IX and XII are linked with some types of cancers as they are overexpressed by tumor hypoxia, and have become a good target for anti-cancer drug design. Moreover, a sulfonamide derivative called SLC-0111, a selective CA IX/XII inhibitor is in Phase II clinical trials for the treatment of primary tumors/metastases, which overexpress these enzymes [5]. 

CA inhibitors (CAIs) targeting mammalian CAs, are used as diuretics, antiglaucoma, antiepileptic, or antiobesity agents for decades [4,6,7,8]. These diverse applications are due to the different distribution of the 15 different hCA isoforms and being involved in critical physiological and pathological processes [9,10,11,12]. On the other hand, abnormal levels or activities of these enzymes have been often related to various human diseases some of which have been clinically exploited and validated as therapeutic targets for the treatment or prevention of different pathologies such as glaucoma, neurological disorders, epilepsy and more recently in cancer [13]. In this context, many efforts are made to discover novel and selective Carbonic Anhydrase Inhibitors (CAIs) leading compounds with potential biomedical applications [14].

In this context, many efforts are made to discover novel and selective Carbonic Anhydrase Inhibitors (CAIs) lead compounds with potential biomedical applications without the side effects that are present in marketed drugs to date [14].

Griseofulvin and usnic acid derivatives attracted the attention of the scientific community due to their wide range of biological activities, such as antimicrobial [15,16,17,18], anticancer [19,20], antiviral [21,22,23] hypoglycemic [24], cytotoxic [18], anticholinergic [25], antioxidant [25], anti-Toxoplasma gondii [26]. Furthermore, it should be mentioned the important role of sulfonamide derivatives. The variety of their biological activities is very wide. They possess antimicrobial [27,28,29], anticancer [30,31,32], anti-inflammatory [33,34], antioxidant [35], antidiabetic [36,37], antimalarial [31,38], dihydrofolate reductase (DHFR) inhibitory [39], and CA inhibitory [40,41,42] activities. Moreover, they play a significant role in CA inhibition, since the sulfonamide group acts as an efficient zinc-binding moiety [43].

**Figure 1:**
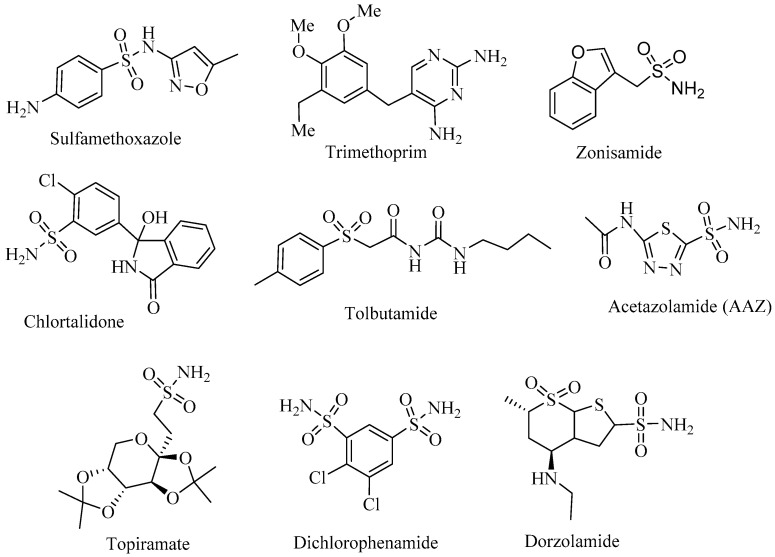
The structure of approved sulfonamide drugs.

There are many drugs on the market incorporating a sulfonamide moiety. Some examples are: the antibiotics sulfamethoxazole (used as cotrimoxazole in combination with trimethoprim), chlortalidone a thiazide-like diuretic, tolbutamide –potassium channel blocker used as oral hypoglycemic medication, zonisamide, a drug for the treatment of epilepsy and Parkinson disease, as well as acetazolamide, carbonic anhydrase inhibitor, topiramate, a sulfamate drug used also to treat epilepsy and migraine, dichlorophenamide, carbonic anhydrase inhibitor used to treat acute angle closure glaucoma, dorzolamide, used to treat high pressure inside the eye, including in cases of glaucoma, and many others [44], (Figure 1).

Previously, we published an article [16,17] on the antimicrobial activity of new derivatives of griseofulvin and usnic acid. According to PASS prediction [45] these derivatives showed carbonic anhydrise inhibitory activity with Pa up to 0.646 and 0.572 for antibacterial and antifungal activities, respectively. On the other hand, as already mentioned above, the sulfonamide fragment is part of a large number of antibiotic drugs, as well as in the composition of many molecules that are carbonic anhydrase inhibitors [40,41,42,43]. The idea of choosing derivatives of griseofulvin and usnic acid containing sulfonamide fragments is based on the so-called search on hybrid molecules containing simultaneously modified natural backbone and sulfonamide moiety with potential properties as specific carbonic anhydrase inhibitors and antibiotic targets. Consequently, herein we present the synthesis of griseofulvin and usnic acid sulfonamide derivatives (**1a**–**j**,**4a**,**b**) and the evaluation of their inhibitory activity against three human CAs (I, II, IX) as well as β and γ CA from different bacterial and one fungal strains.

## 2. Results and Discussion

### 2.1. Preliminary Docking Studies

Computational methods are an important part of the drug design process and they are presently used to get a deeper understanding of the drug-enzyme interactions. Molecular docking studies were performed in a series of designed griseofulvin and usinc aisid derivatives bearing sulfamoyl moiety. As presented in Table S1 from all compounds only the ones highlighted in red exhibited good to excellent binding energy and were selected for further studies.

### 2.2. Prediction of Toxicity

Predicting the toxicity of a compound is a critical step in the development of new drug candidates, making in silico toxicity studies a faster and cheaper procedure than in vivo animal toxicity testing or in vitro testing in cell lines. It also helps significantly reduce the number of animals used in experimental assays. There are several online programs that access toxicities that use in silico models to predict mean lethal dose, carcinogenicity, mutagenicity, and more.

The Pro-Tox II web server [46] predicts the mean lethal dose (LD50) in rodents. According to this program, all compounds can be classified into six GHS (Globally Harmonized System of Classification and Labeling of Chemicals) Categories [47] according to their toxicity and LD50 value.

Toxicity classes are defined according to the globally harmonized system of classification of labeling of chemicals (GHS). LD50 values are given in [mg/kg]:

▪ Class I: fatal if swallowed (LD50 ≤ 5)
▪ Class II: fatal if swallowed (5 < LD50 ≤ 50)
▪ Class III: toxic if swallowed (50 < LD50 ≤ 300)
▪ Class IV: harmful if swallowed (300 < LD50 ≤ 2000)
▪ Class V: may be harmful if swallowed (2000 < LD50 ≤ 5000)
▪ Class VI: non-toxic (LD50 > 5000)

According to prediction, the compounds found to be at category IV and V with LD_50_ ranging from 838 to 5000 mg/kg. All compounds were predicted to be non-cytotoxic, non-carcinogenic, non-hepatotoxic, and non-mutagenic Especially compounds **4a** and **4b** predicted as the most non-toxic of all with the best-predicted profile (Table S2).

### 2.3. Chemistry

**Scheme 1:**
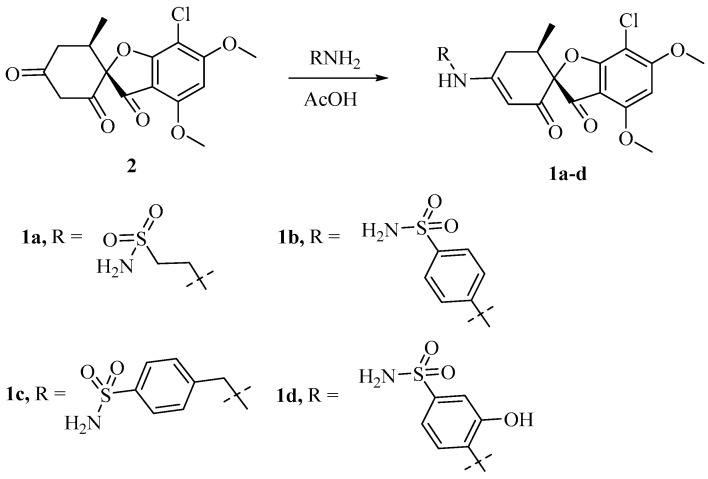
Synthesis of compounds **1a**–**d**.

Griseofulvin derivatives **1a**–**d** were obtained by the reaction of *griseofulvic* acid **2** and corresponding amine in refluxing acetic acid. The starting material *griseofulvic* acid **2** was synthesized from griseofulvin by known procedures [48]. This method allows one to prepare the target products **1a**–**d** in 42–66% yields (Scheme 1).

**Scheme 2:**
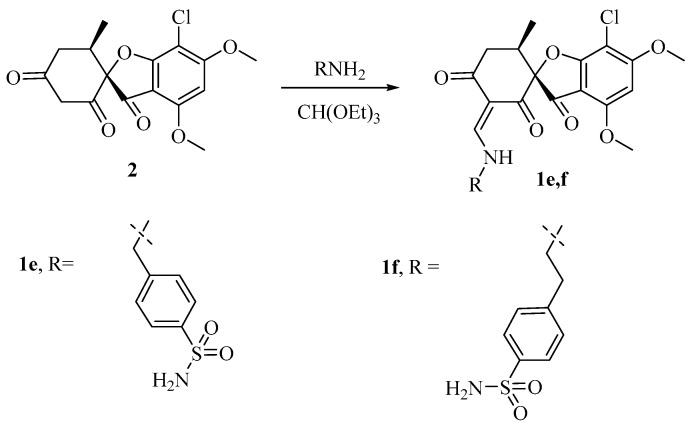
Synthesis of compounds **1e**,**f**.

The griseofulvin derivatives **1e**,**f** was synthesized by condensation of *griseofulvic* acid **2** with the corresponding amine. Excess triethyl orthoformate was used as solvent wherein the final products were obtained in 47–51% yields (Scheme 2). 

**Scheme 3:**
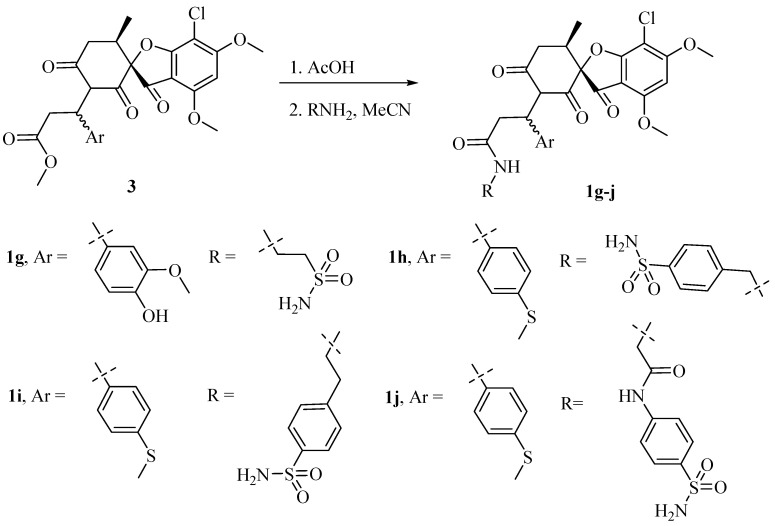
Synthesis of compounds **1g**–**j**.

The griseofulvin amide derivatives **1g**–**j** were synthesized from corresponding esters **3**. Starting *griseofulvic* esters **3** were prepared via a multicomponent reaction of *griseofulvic* acid, aldehydes, and Meldrum’s acid using a previously described protocol [22]. The obtained products were synthesized in 68–86% yields (Scheme 3). Note that starting compounds **3** and target amides **1g**–**j** exist as a mixture of diastereomers.

**Scheme 4:**
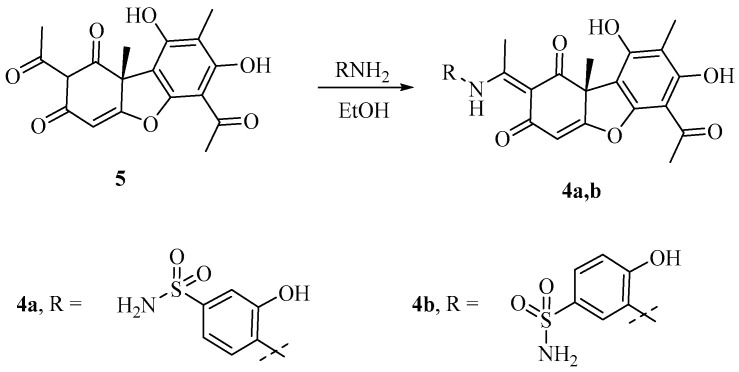
Synthesis of compounds **4a**,**b**.

The usnic acid derivatives **4a**,**b** were obtained by the previously described method from (*R*)-usnic acid **5** and appropriate amines in refluxing ethanol [17]. The yields of synthesized products were 54–67% (Scheme 4).

The structure of all compounds (Table 1) was characterized by ^1^H-NMR, ^13^C-NMR, and elemental analysis and presented in the experimental part. 

### 2.4. Evaluation of CA Inhibitory Activity

All compounds **1a**–**j**, **4a**,**b** were evaluated for their inhibitory activity against three human CA isoforms, namely: hCA I, hCA II, hCA IX, and CAs from some bacterial and fungal pathogenic species, such as *Malassezia globosa* (β-CA from a fungus involved in dandruff formation [49]), MgCA, as well as β- and γ-CAs from the following bacteria: *Porphyromonas gingivalis* (PgiCA); *Streptococcus mutans* (SmuCA); *Burkholderia pseudomallei* (BpsCA) and *Colwellia psychrerythraea*, a non-pathogenic Antarctic bacterium (CpsCA) [50,51,52,53,54]. The results are shown in Table 2. The evaluation revealed that all compounds exhibited carbonic anhydrase inhibitory activity against all isoforms tested, but with different ranges of inhibition constants. For example, in the case of hCA I the K_i_ value of tested compounds arethe in range from 15.3 to 2986 nM, while for hCA II and hCA IX the Ki ranged from 4.9 to 4052 nm and 22.5 to 709 nm, respectively. According to the data in Table 2 it is obvious that tested compounds are more potent against the hCA I isoform, with seven out of twelve compounds being more active inhibitors than acetazolamide, used as a reference drug. The compound’s order of activity can be presented as follows: **1i** > **1j** > **1d** > **1h** > **1c** > **4b** > **1b** > **1e** > **1f** > **4a** > **1a** > **1g**. The highest activity was achieved by compound **1i** with K_i_ value of 15.3 nM compared with **AAZ** (K_i_ = 250 nM). The lowest activity was observed for compound **1g** with a K_i_ value of 2986 nM.

As far as the inhibitory activity of compounds against the hCAII isoform is concerned five compounds (**1d**, **1h**, **1i**, **1j**, and **4a**) appeared to be more potent than the reference drug. According to the activity order of compounds against hCA II which is: **1d** > **1j** > **1h** > **4a**> **1c** > **1b** > **1f** > **1e**> **4b** > **1g** demonstrating compound **1d** is the most active with K_i_ 4.9 nM and a selectivity index (SI) towards hCA I and hCA IX of 5.88 and 6.00 respectively. On the other hand, derivative **1g** showed the lowest activity with a K_i_ of 4052 nM. It should be mentioned that the most selective derivative among all compounds tested was **4a** with a SI towards hCA I and hCA IX of 43.9 and 7.96, respectively.

Regarding the inhibition activity against the hCA IX isoform, in general, all compounds showing moderate to low activity in the following order: **1j** > **1i** > **1e** > **1d** > **1f** > **1a** > **4a** > **1h** > **1c** > **1b** > **4b** > **1g.** Thus, the most potent appeared to be compound **1j** with K_i_ of 22.5 nM, followed by **1i** (K_i_ of 23.3 nM), while the lowest activity was observed for compound **1g** with K_i_ of 709.6 nM. The comparison of the activity towards three human CA isoforms revealed that compounds **1d**, **1i**, and **1j** were among the most active against all isoforms, whereas compound **1g** showed the lowest activity in all cases.

The study of structure-activity relationships revealed that the presence of (4-(methylthio)phenyl)-N-(4-sulfamoylphenethyl)propionamide (**1i**) in position 3 of griseofulvin seems to be beneficial for hCA I inhibitory activity. Replacement of N-(4-sulfamoylphenethyl)acetamide (**1i**) by N-(2-oxo-2-((4-sulfamoylphenyl)amino) ethyl)propionamide resulted in a compound **1j** with decreased activity. The introduction of 4-amino-3-hydroxybenzenesulfonamide group instead of 2-acetamido-N-(4-sulfamoylphenyl)acetamide in position 4 of griseofulvin led to less potent compound **1d**, while the presence of N-(4-sulfamoylbenzyl)propionamide in position 3 of griseofulvin (**1h**) decreased more the activity compared to compound **1d**. Nevertheless, all these compounds were among the most active ones. On the other hand, the replacement 4-methylthiophenyl group by 4-hydroxy-3-methoxyphenyl **(9**) was detrimental to hCA I inhibition.

Regarding the structure-activity relationships in the case of the hCA II isoform, despite the first four the most active compounds, as already mentioned being the same, the influence of substituent’s is different. Thus, the presence of 4-amino-3-hydroxybenzenesulfonamide in position 4 of the main core of compound (**1d**) was favorable for hCA II inhibitory activity, while the introduction of (4-(methylthio)phenyl)-N-(4-sulfamoylphenethyl)propionamide to position 3 of griseofulvin core led to compound **1i**, which exhibited the lowest activity among the four (**1d**, **1h**, **1i**, **1j**) most active ones. The presence of N-(2-oxo-2-((4-sulfamoylphenyl)amino)ethyl)propionamide (**1j**) showed the same influence on activity as in the case of hCA I, being the second active one, while replacement of 4-methylthiophenyl group by 4-hydroxy-3-methoxyphenyl (**1g**) in position 3 of griseofulvin moiety was detrimental as in case of hCA I.

According to a structure-activity relationship study for the hCA IX isoform the presence of N-(2-oxo-2-((4-sulfamoylphenyl)amino)ethyl)propionamide (**1j**) had a very positive influence on inhibitory activity, while its replacement by 4-(methylthio)phenyl)-N-(4-sulfamoylphenethyl)propionamide led to second more active compound (**1i**). Introduction at position 3 of the main core of compound 4-((vinylamino)methyl)benzenesulfonamide substituent resulted in less active compound (**1e**), while the presence of 4-amino-3-hydroxybenzenesulfonamide in position 4 decreased more the activity leading to compound **1d**.

From all mentioned above it can be concluded that the same substitution at position 3 of the main core of compounds (1′*S*,6′*R*)-7-chloro-2′-hydroxy-4,6-dimethoxy-6′- methyl-3′-(4-(methylthio)benzyl)-3H-spiro[benzofuran-2,1′-cyclohex [2]ene]-3,4′-dione play different role dependent on hCA isoform.

Furthermore, we investigated the activity of our compounds towards three beta (*PgiCAβ*, *SmuCA*, and *MgCA*) and three gamma CAs (*BpsCAγ*, *PgiCAγ*, *Csp*CA*γ*) from different microorganisms. It was found that the compounds showed inhibitory activity against all the bacterial CAs examined but to carrying extents. Only in the case of *Pgi*CA*γ, Csp*CA*γ* and *St.mutans* some compounds excited the activity of reference drug. Thus, compound **1a** exhibited excellent activity against *Pgi*CA*γ* with K_i_ at 9.1 nM and SI 8.5 towards *Pgi*CAβ being 3.5 fold more active than acetazolamide. Three compounds, **1e**, **1g** and **1j** were found to be more potent than the reference drug against *Cps*CAγ. Among them, the highest activity was achieved for compound **1j** with K_i_ at 176.8 nM compared to acetazolamide (K_i_ of 502 nM). This compound appeared to be selective towards *Pgi*CAγ, *S. mutans*, and *Bps*CAγ with SI 13, 6.9, and 4, respectively. Finally, one compound **1b** (K_i_ at 293.7 nM) was the only which exceeded the activity of the reference drug against *S. mutans* (K_i_ of 344 nM and SI 3 towards all other bacterial strains.

In addition, our compounds showed very good activity in the case of the fungal isoform from *Malassezia globosa* (MgCA), being all twelve compounds more potent than acetazolamide with K_i_ in the range of 475.6–4346 nM compared to reference drug (K_i_ of 40,000 nM). The order of activity can be presented as: **1b** > **1a** > **1c** > **1d** > **1e** > **1i** > **1j** > **4b** > **1g** > **4a** > **1f** > **1h**, with the best activity exhibiting by compound **1b** with K_i_ at 475.6 nM followed by compound **1a** (K_i_ of 544 nM). The less potent appeared to be compound **1h** with Ki at 4346 nM. The comparison of activity towards MgCA with hCA isoforms revealed that among the most active compounds, only one is common (**1d**). Besides, compound **1h** is between the four most active compounds in the case of hCA isoforms ranking from first to fourth place in the activity order, whereas in the case of MgCA it is the less active one.

According to structure-activity relationship studies the presence of the 4-aminobenzensulfonamide group in the main core (**1b**) is beneficial for Mg CA inhibitory activity, while its replacement by 4-(aminomethyl)benzenesulfonamide decreased activity leading to compound **1c**. The introduction of 4-amino-3-hydroxybenzenesulfonamide resulted in a less active compound (**1d**) compared to the previous one (**3**), while the presence of N-(4-sulfamoylbenzyl)acetamide (**1h**) substituent played a negative role to activity.

Thus, according to obtained results, it is obvious that substituents and their position play an important role in CA inhibitory activity, human and microbial.

### 2.5. Molecular Docking Studies in Human CAs Isoforms

As representatives of the whole set of compounds, **1c**, **1g**, **1f**, and **1k** were chosen for docking studies in order to predict possible inhibition mechanisms. 

All human CAs isoforms contain conserved residues His94, His96, and His119 in their active sites. In this way, these residues act as zinc ligands. In addition, all isoforms have two additional conserved residues at the active site, Thr199 and Glu105, that serve as ‘gatekeepers’ [55,56,57]. Nevertheless, these isoforms differ mainly between their middle and exit residues in the active site cavity.

Molecular docking results for the tested compounds on hCA I, II, and IX isoforms are shown in Table 3. Based on these results, all compounds chelating the Zn (II) ion are anions (negative nitrogen of the sulfonamide group) that bind the enzymes in the same manner [57].

Docking results reveal that the selectivity profiles and inhibition modes of some compounds depend on variations in enzyme active sites. The nature of the amino acids in the enzyme’s active site as well as the substitution of each compound determines the conformation that compounds adopt within the active site of the enzyme and how they interact with it.

**Figure 2:**
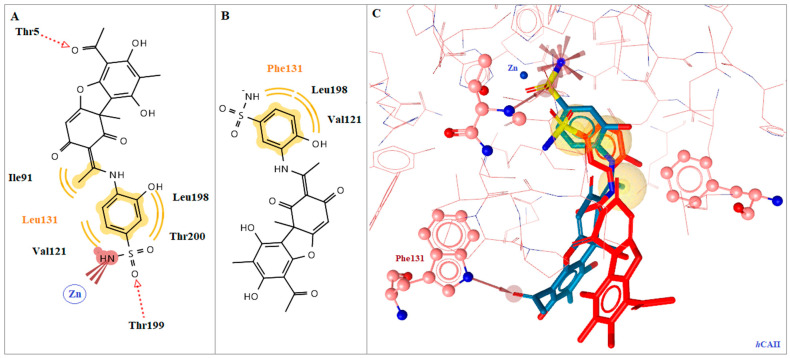
(**A**) 2D interaction diagram of compound **4a** docking pose interactions with the key amino acids in hCA II, (**B**) 2D interaction diagram of compound **4b** docking pose interactions with the key amino acids in hCA II. (**C**) Superposition of compound **4a** (blue) bound to hCA II in comparison to compound **4b** (red) to hCA II, with specific residues labeled. Active site zinc is shown as a blue sphere, red dotted arrows indicate H-bonds, and yellow spheres hydrophobic interactions.

Taking all these into account, comparing the docking poses in the *h*CA II enzyme of compounds **4a** and **4b** with Ki values for the *h*CA II enzyme of 8.4nM and 755.3 nM respectively, we can say that the para substitution of sulphonamide group in compound **4a** plays an important role in the inhibition profile of this compound compared to compound **4b.** *h*CA II enzyme has a hydrophobic residue Phe131 in the active site that provides a bulky environment for the compound to freely enter the active site. Compound **4a** having a para substitution of sulphonamide group provides a more line structure that can have the flexibility and enables it to avoid the steric hindrance of the bulky residue Phe131 *h*CA II isoform, increasing the inhibition potency (Figure 2).

As it is illustrated in Figure 2 this compound (**4a**) inserts to the active site of the enzyme freely, the negative nitrogen of the sulphonamide group chelates the Zn (II) ion and forms hydrogen bonds. Moreover, the oxygen atom of the sulphonamide group forms a hydrogen bond with residue Thr199 (distance 1.73). Furthermore, the benzene moiety is interacting hydrophobically with residues Val121, Leu131, Thr200, and Leu198. These interactions further stabilize the complex and explain its high inhibition potency (Figure 2A).

On the other hand, compound **4b** probably because of the presence of meta substitution in the benzene ring cannot fully enter the active site of the enzyme and reach the Zn ion to chelate resulting in its low inhibition potency (Figure 2B,C).

**Figure 3:**
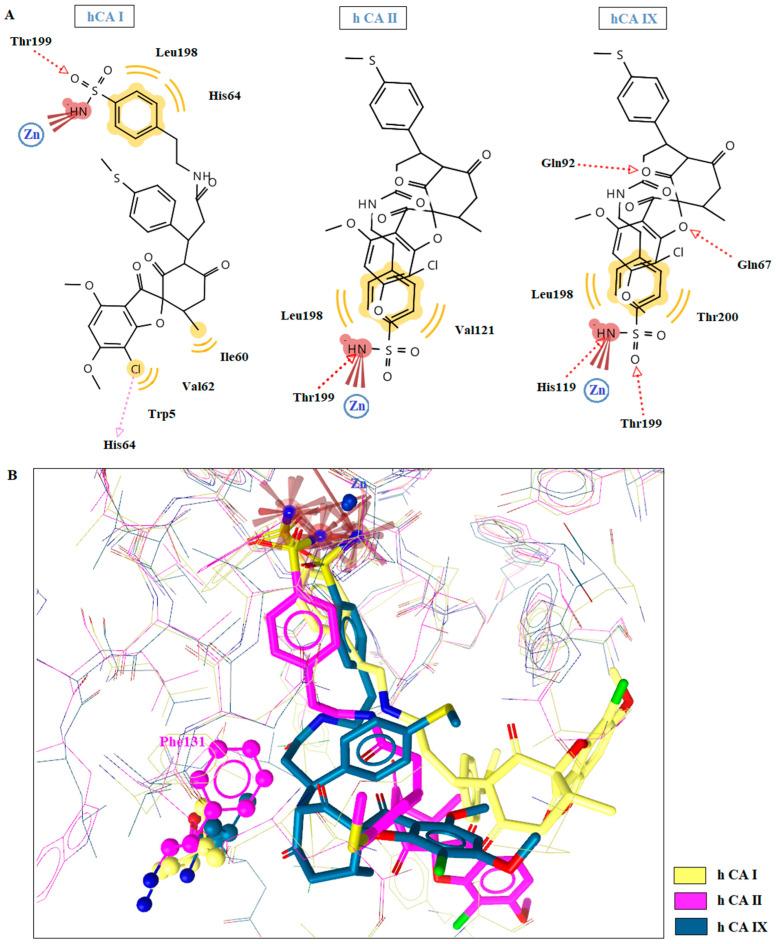
(**A**) 2D interaction diagram of compound **1i** docking pose interactions with the key amino acids in hCA I, hCA II, and hCA IX (**B**) Superposition of compound **1i** (yellow)bound to hCA I in comparison with its binding to hCA II (magenta) and hCA IX (blue) isoforms. Active site zinc shown as blue sphere, red dotted arrows indicate H-bonds, pink arrow halogen bond and yellow spheres hydrophobic interactions.

The flexible structure of compound **4i** can also explain its inhibition potency towards hCA I, hCA II, and hCA IX enzymes with Ki values of 15.3, 8.1, and 23.3 nM respectively. Indeed, the superposition of this compound bound to hCA I in comparison to hCA II and hCA IX (Figure 3) shows that it can adopt a conformation that favors the interactions with all active sites of the isoforms, avoiding the steric hindrance of the bulky residue Phe131 of hCA II isoform and increasing the stability of each complex and subsequently the inhibition potency of the compound.

In particular, in all isoform structures, the negative nitrogen of the sulphonamide group chelates the Zn (II) ion and forms hydrogen bonds (Figure 3A). In all isoforms, the one oxygen atom of the sulphonamide group forms a hydrogen bond with residue Thr199. Furthermore, in isoform hCA I, the Cl atom of the benzene ring is forming a halogen bond with residue His64. Additionally, the benzene ring is interacting hydrophobically with Val121 and Leu198 (Figure 3A). In isoform hCA IX there are except the aforementioned H-bond with residue Thr199, another three hydrogen bonds formed with residues His119, Gln67, and Gln92. These interactions can probably explain the high ki value of compound **1i** against all isoforms and especially its superiority over AAZ in isoform hCA IX. The same conclusion can be made and for compounds **1h** and **1j** with a similar structure to compound **1i**.

### 2.6. Drug Likeneess

**Figure 4:**
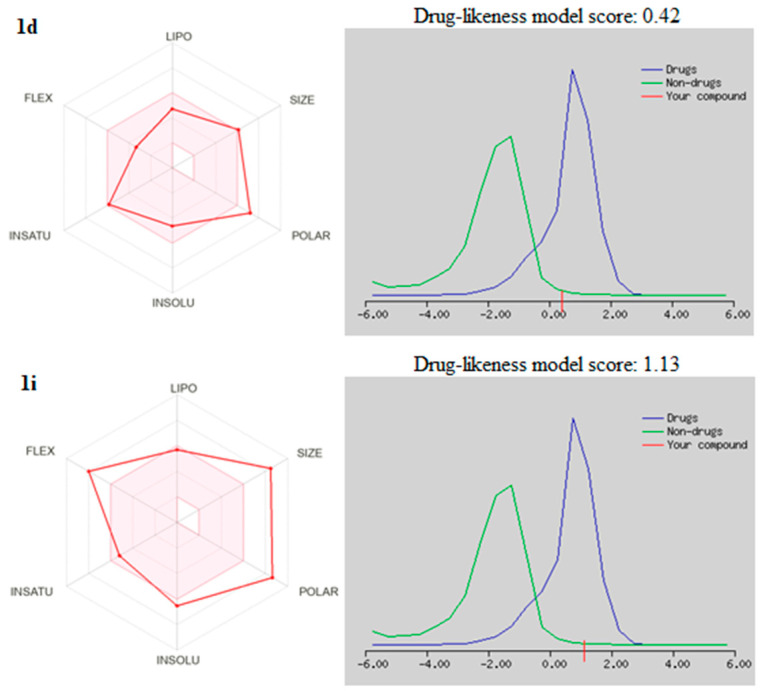
Drug-likeness model and bioavailability Radar of the compounds **1d** and **1i**. The pink area represents the optimal range for each property for oral bioavailability, (Lipophilicity (LIPO): XLOGP3 between −0.7 and +5.0, Molecular weight (SIZE): MW between 150 and 500 g/mol, Polarity (POLAR) TPSA between 20 and 130 Å^2^, Solubility (INSOLU): log S not higher than 6, Saturation (INSATU): fraction of carbons in the sp3 hybridization not less than 0.25, and Flexibility (FLEX): no more than 9 rotatable bonds.

All tested compounds were evaluated for their Drug-likeness and bioavailability scores and the results of the prediction are shown in Table 4. According to prediction, the bioavailability score of most of the compounds was about 0.55 except for compounds **1g**, **1h**, **1j**, **4a** and **4b** with 0.17 values Only five compounds showed 2 violations of Lipinski’s rule of five and in combination with their excellent Drug-likeness scores ranging from -0.13 to 1.24, it can be concluded that they have good oral bioavailability (Figure 4).

## 3. Materials and Methods

### 3.1. Chemistry

#### 3.1.1. Synthesis of *Griseofulvin* Derivatives **1a**–**d**

The mixture of *griseofulvic* acid **2** (0.68 g, 2 mmol) and corresponding amine (2.2 mmol) (0.18 g, 2.2 mmol of anhydrous sodium acetate was added in case of amine hydrochloride) in AcOH (7 mL) was refluxed 7 h. Next, the obtained solution was evaporated and the residue was dissolved in MeOH (5 mL) and then H_2_O (5 mL) was added to the solution. The precipitate formed was collected by filtration, washed with 30% aqueous MeOH (3*7 mL), and dried to afford pure compounds **1a**–**d**.

2-(((2*S*,6′*R*)-7-chloro-4,6-dimethoxy-6′-methyl-2′,3-dioxo-3H-spiro[benzofuran-2,1′-cyclohexan]-3′-en-4′-yl)amino)ethane-1-sulfonamide **1a**.

Yield 42%, m.p. 185–187 °C.^1^H NMR (300 MHz, DMSO-*d*_6_) δ 7.43 (br. s, 1H, NH), 6.81 (br. s, 2H, NH_2_), 6.37 (s, 1H, CH), 4.98 (s, 1H, CH), 4.03 (s, 3H, OCH_3_), 3.93 (s, 3H, OCH_3_), 3.54–3.50 (m, 2H, CH_2_), 3.22–3.18 (m, 2H, CH_2_), 3.04–2.98 (m, 1H, CH), 2.72–2.65 (m, 1H, CH), 2.41–2.36 (m, 1H, CH), 0.92 (d, *J* = 6.5 Hz, 3H, CH_3_). ^13^C NMR (75 MHz, DMSO-*d*_6_) δ 192.77 (C = O), 185.03 (C = O), 169.55 (C-NH), 164.53, 164.23 (C-OCH_3_), 157.68, 105.31, 95.51, 94.86, 92.74, 91.04, 57.86, 56.83(C-OCH_3_), 56.47, 52.64, 37.87, 35.23 (C-CH_3_), 32.19, 14.93 (CH_3_).). Anal. Calcd. for C_18_H_21_ClN_2_O_7_S(%).C, 48.59; H, 4.76; N, 6.30%. Found: C, 48.39; H, 4.80; N, 6.38%.

4-(((2*S*,6′*R*)-7-chloro-4,6-dimethoxy-6′-methyl-2′,3-dioxo-3H-spiro[benzofuran-2,1′-cyclohexan]-3′-en-4′-yl)amino)benzenesulfonamide **1b**.

Yield 66%, m.p. 234–236 °C.^1^H NMR (300 MHz, DMSO-*d*_6_) δ 9.44 (s, 1H, NH), 7.83 (d, *J* = 8.6 Hz, 2H, 2CH), 7.36 (d, *J* = 8.6 Hz, 2H, 2CH), 7.18 (s, 2H, NH_2_), 6.39 (s, 1H, CH), 5.50 (s, 1H, CH), 4.03 (s, 3H, OCH_3_), 3.93 (s, 3H, OCH_3_), 3.24–3.13 (m, 1H, CH), 2.82–2.75 (m, 1H, CH), 2.68–2.61 (m, 1H, CH), 0.97 (d, *J* = 6.5 Hz, 3H, CH_3_). ^13^C NMR (75 MHz, DMSO-*d*_6_) δ 192.16 (C = O), 186.65 (C = O), 169.44, (C-O) 164.38 (C-OCH_3_), 162.00, 157.81, 142.07, 140.19, 127.65, 122.99, 105.07, 96.84 (C-Cl), 95.57, 94.77, 91.22, 56.89(O-CH_3_), 56.47 (O-CH_3_), 32.61 (C-CH_3_), 18.99, 14.85 (CH_3_). Anal. Calcd. for C_22_H_21_ClN_2_O_7_S(%).C, 53.61; H, 4.29; N, 5.68%. Found: C, 53.35; H, 4.47; N, 5.76%.

4-((((2*S*,6′*R*)-7-chloro-4,6-dimethoxy-6′-methyl-2′,3-dioxo-3H-spiro[benzofuran-2,1′-cyclohexan]-3′-en-4′-yl)amino)methyl)benzenesulfonamide **1c**.

Yield 57%, m.p. 202–204 °C.^1^H NMR (300 MHz, DMSO-*d*_6_) δ 8.01 (br. s, 1H, NH), 7.83 (d, *J* = 8.0 Hz, 2H, 2CH), 7.48 (d, *J* = 8.0 Hz, 2H, 2CH), 7.12 (br. s, 2H, NH_2_), 6.36 (s, 1H, CH), 4.83 (s, 1H, CH), 4.40 (d, *J* = 5.6 Hz, 2H, CH_2_), 4.02 (s, 3H, OCH_3_), 3.91 (s, 3H, OCH_3_), 3.15–3.00 (m, 1H, CH), 2.71–2.62 (m, 1H, CH), 2.52–2.48 (m, 1H, CH), 0.93 (d, *J* = 6.6 Hz, 3H, CH_3_). ^13^C NMR (75 MHz, DMSO-*d*_6_) δ 192.16 (C = O), 186.65 (C = O), 169.44 (C-NH), 164.38, 162.00 (C-OCH_3_), 157.81 (C-OCH_3_), 142.07 (C-SO_2_), 140.19, 127.65 (2C), 122.99 (2C), 105.07, 96.84, 95.57, 94.77, 91.22, 57.90 (O-CH_3_), 56.89 (O-CH_3_), 56.47, 39.86, 32.61 (C-CH_3_), 18.99, 14.85 (C-CH_3_). Anal. Calcd. for C_23_H_24_ClN_2_O_7_S(%).C, 53.49; H, 4.57; N, 5.53%. Found: C, 53.50; H, 4.47; N, 5.6%.

4-(((2*S*,6′*R*)-7-chloro-4,6-dimethoxy-6′-methyl-2′,3-dioxo-3H-spiro[benzofuran-2,1′-cyclohexan]-3′-en-4′-yl)amino)-3-hydroxybenzenesulfonamide **1d**.

Yield 64%, m.p. 255–257 °C.^1^H NMR (300 MHz, DMSO-*d*_6_) δ 10.28 (s, 1H, NH), 8.79 (s, 1H, OH), 7.42 (s, 1H, CH), 7.31 (s, 2H, 2CH), 7.10 (s, 2H, NH_2_), 6.37 (s, 1H, CH), 5.07 (s, 1H, CH), 4.03 (s, 3H, OCH_3_), 3.93 (s, 3H, OCH_3_), 3.22–3.01 (m, 1H, CH), 2.78–2.67 (m, 2H, 2CH), 0.96 (d, *J* = 5.9 Hz, 3H, CH_3_). ^13^C NMR (75 MHz, DMSO-*d*_6_) δ 192.54 (C = O), 185.84 (C = O), 169.51 (C-O), 164.28 (C-OCH_3_), 163.84 (C-NH), 157.73(C-OCH_3_), 151.68, 142.60 (C-S), 128.81, 127.03, 117.21, 114.09, 105.23, 95.53, 94.82, 91.10, 57.88(O-CH_3_), 56.85 (O-CH_3_), 35.32 (C-CH_3_), 32.05, 14.96 (CH_3_). Anal. Calcd. for C_22_H_21_ClN_2_O_8_S(%).C, 51.92; H, 4.16; N, 5.50%. Found: C, 51.89; H, 4.29; N, 5.76%.

#### 3.1.2. Synthesis of *Griseofulvin* Derivatives **1e**,**f**

The mixture of *griseofulvic* acid **2** (0,68 g, 2 mmol) and corresponding amine (2.2 mmol) (0.22 g, 2.2 mmol of Et_3_N was added in case of amine hydrochloride) in triethyl orthoformate (7 mL) was refluxed for 6 h. Next, the obtained solution was evaporated and the residue was dissolved in MeOH (5 mL) and then H_2_O (5 mL) was added to the solution. The precipitate formed was collected by filtration, washed with 30% aqueous MeOH (3*7 mL), and dried to afford pure compounds **1e**,**f**.

4-((((2*R*,2′*R*)-7-chloro-4,6-dimethoxy-2′-methyl-3,4′,6′-trioxo-3H-spiro[benzofuran-2,1′-cyclohexan]-5′-ylidene)methyl)amino)methyl)benzenesulfonamide **1e**.

Yield 47%, m.p. 199–201 °C. A mixture of E- and Z-isomers. ^1^H NMR (300 MHz, DMSO-*d*_6_) δ 11.53 (br. s, 0.5H, NH), 11,17 (br. s, 0.5H, NH), 8.35 (d, *J* = 14.0 Hz, 0.5H, CH), 8.24 (d, *J* = 13.8 Hz, 0.5H, CH), 7.83 (d, *J* = 8.0 Hz, 2H, 2CH), 7.51 (d, *J* = 8.0 Hz, 2H, 2CH), 7.21 (br. s, 2H, NH_2_), 6.40 (s, 1H, CH), 4.78 (s, 2H, CH_2_), 4.03 (s, 3H, OCH_3_), 3.93 (s, 3H, OCH_3_), 3.08–2.91 (m, 1H, CH), 2.85–2.68 (m, 1H, CH), 2.52–2.47 (m, 1H, CH), 0.91 (d, *J* = 6.4 Hz, 3H, CH_3_). ^13^C NMR (75 MHz, DMSO-*d*_6_) δ 194.24 (C = O), 191.75 (C = O), 188.46 (C = O), 165.09, 165.04, 163.55 (C-OCH_3_), 159.48 (C-OCH_3_), 143.10, (C-SO_2_) 140.41, 127.01 (2C), 126.43 (2C), 105.48, 102.01, 101.52, 92.70, 88.47, 56.49 (C-OCH_3_), 56.39 (C-OCH_3_), 52.19, 40.82, 31.64, 14.34 (C-CH_3_). Anal. Calcd. for C_24_H_23_ClN_2_O_8_S(%).C, 53.88; H, 4.33; N, 5.24%. Found: C, 53.89; H, 4.37; N, 5.31%.

4-(2-((((2*R*,2′*R*)-7-chloro-4,6-dimethoxy-2′-methyl-3,4′,6′-trioxo-3H-spiro[benzofuran-2,1′-cyclohexan]-5′-ylidene)methyl)amino)ethyl)benzenesulfonamide **1f**.

Yield 51%, m.p. 193–195 °C. A mixture of E- and Z-isomers. ^1^H NMR (300 MHz, DMSO-*d*_6_) δ 11.32 (br. s, 0.5H, NH), 10,90 (br. s, 0.5H, NH), 8.21 (d, *J* = 14.1 Hz, 0.5H, CH), 8.05 (d, *J* = 13.7 Hz, 0.5H, CH), 7.82–7.72 (m, 2H, 2CH), 7.48–7.36 (m, 2H, 2CH), 7.09 (s, 2H, NH_2_), 6.41 (s, 1H, CH), 4.05 (s, 3H, OCH_3_), 3.94 (s, 3H, OCH_3_), 3.81–3.73 (m, 2H, CH_2_), 3.05–2.94 (m, 3H, CH_2_ + CH), 2.77–2.68 (m, 1H, CH), 2.51–2.33 (m, 1H, CH), 0.92 (d, *J* = 6.3, 3H, CH_3_). ^13^C NMR (75 MHz, DMSO-*d*_6_) δ 185.83 (3C, C = O), 164.55 (4C), 129.71 (2C), 126.27 (4C), 95.54, 94.81, 94.49, 91.31 (2C), 57.97 (O-CH_3_), 56.93 (O-CH_3_), 51.26 (CH_2_-NH), 33.48 (3C), 15.00 (CH_3_). Anal. Calcd. for C_25_H_25_ClN_2_O_8_S(%).C, 54.69; H, 4.59; N, 5.10%. Found: C, 54.59; H, 4.65; N, 5.00%.

#### 3.1.3. Synthesis of *Griseofulvin* Derivatives **1g**–**j**

The solution of corresponding ester **3** (2 mmol) in AcOH (8 mL) was refluxed for 5 h. Then obtained solution was evaporated, the residue was dissolved in MeCN (7 mL) and corresponding amine (2.2 mmol) was added (0.22 g, 2.2 mmol of Et_3_N was used in case of amine hydrochloride). The reaction mixture was refluxed for 8 h, then the solution was evaporated. The residue was dissolved in MeOH (5 mL), HCl_conc_ (1 mL) and H_2_O (25 mL) were added to the reaction mixture. The precipitate formed was collected by filtration, washed with 2% HCl solution (3*10 mL) and H_2_O (3*10 mL), and dried to afford pure compounds **1g**–**j**.

3-((2*S*,2′*R*)-7-chloro-4,6-dimethoxy-2′-methyl-3,4′,6′-trioxo-3H-spiro[benzofuran-2,1′-cyclohexan]-5′-yl)-3-(4-hydroxy-3-methoxyphenyl)-N-(2-sulfamoylethyl)propenamide **1g**.

Yield 68%, m.p. 204–206 °C.^1^H NMR (300 MHz, DMSO-*d*_6_) δ 8.17 (br. s, 1H, OH), 7.60 (br. s, 1H, NH), 6.75 (s, 1H, CH), 6.67–6.55 (m, 4H, 2CH + NH_2_), 6.37 (s, 1H, CH), 4.53 (t, *J* = 7.6 Hz, 1H, CH), 4.02 (s, 3H, OCH_3_), 3.92 (s, 3H, OCH_3_), 3.74 (s, 3H, OCH_3_), 3.41–3.35 (m, 2H, CH_2_), 3.16–2.46 (m, 7H, 5CH+CH_2_), 0.91 (d, *J* = 6.3 Hz, 3H, CH_3_). ^13^C NMR (75 MHz, DMSO-*d*_6_) δ 201.35 (C = O), 196.73 (2C, C = O), 173.06 (C = O), 162.91 (C-OCH_3_), 157.12 (C-OCH_3_), 156.72 (C-OCH_3_), 151.24 (C-OH), 149.37, 144.04, 140.28, 127.73, 124.39, 111.00, 106.90, 104.79, 101.64, 90.53, 60.33 (2C), 56.47 (3C), 31.46, 30.46 (2C), 19.00 (2C), 13.49 (C-CH_3_). Anal. Calcd. for C_28_H_31_ClN_2_O_11_S(%).C, 52.62; H, 4.89; N, 4.38%. Found: C, 52.59; H, 4.91; N, 4.34%.

3-((2*S*,2′*R*)-7-chloro-4,6-dimethoxy-2′-methyl-3,4′,6′-trioxo-3H-spiro[benzofuran-2,1′-cyclohexan]-5′-yl)-3-(4-(methylthio)phenyl)-N-(4-sulfamoylbenzyl)propenamide **1h**.

Yield 86%, m.p. 233–235 °C.^1^H NMR (300 MHz, DMSO-*d*_6_) δ 8.18 (t, *J* = 7.1 Hz, 1H, NH), 7.72 (d, *J* = 8.2 Hz, 2H, 2CH), 7.28–7.01 (m, 8H, 6CH+NH_2_), 6.38 (s, 1H, CH), 4.63 (t, *J* = 7.6 Hz, 1H, CH), 4.27 (d, *J* = 7.1 Hz, 2H, CH_2_), 4.03 (s, 3H, OCH_3_), 3.91 (s, 3H, OCH_3_), 3.06–2.87 (m, 2H, 2 CH), 2.65–2.51 (m, 3H, 3CH), 2.44 (s, 3H, SCH_3_), 0.90 (d, *J* = 6.1 Hz, 3H, CH_3_). ^13^C NMR (75 MHz, DMSO-*d*_6_) δ 169.33 (3C, C = O), 164.38, 157.78 (C-OCH_3_), 144.21 (C-OCH_3_), 142.83 (C-S), 134.98 (C-S-CH_3_), 128.73 (2C), 127.55 (4C), 126.16 (2C), 125.96 (4C), 104.76 (2C), 57.91, 56.84 (2C), 42.04 (3C), 35.34 (2C), 15.45 (S-CH_3_) 14.79 (C-CH_3_). Anal. Calcd. for C_33_H_33_ClN_2_O_9_S(%).C, 56.52; H, 4.74; N, 4.00 %. Found: C, 56.50; H, 4.77; N, 4.05%.

3-((2*S*,2′*R*)-7-chloro-4,6-dimethoxy-2′-methyl-3,4′,6′-trioxo-3H-spiro[benzofuran-2,1′-cyclohexan]-5′-yl)-3-(4-(methylthio)phenyl)-N-(4-sulfamoylphenethyl)propenamide **1i**.

Yield 72%, m.p. 192–194 °C.^1^H NMR (300 MHz, DMSO-*d*_6_) δ 7.81–7.62 (m, 3H, 2CH+NH), 7.28 (d, *J* = 8.3 Hz, 2H, 2CH), 7.17 (d, *J* = 8.2 Hz, 2H, 2CH), 7.10–7.01 (m, 4H, 2CH+NH_2_), 6.37 (s, 1H, CH), 4.60 (t, *J* = 7.4 Hz, 1H, CH), 4.03 (s, 3H, OCH_3_), 3.88 (s, 3H, OCH_3_), 3.23 (d, *J* = 6.5 Hz, 2H, CH_2_), 3.01–2.82 (m, 2H, CH_2_), 2.71–2.52 (m, 5H, 5CH), 2.42 (s, 3H, SCH_3_), 0.90 (d, *J* = 6.1 Hz, 3H, CH_3_). ^13^C NMR (75 MHz, DMSO-*d*_6_) δ 169.30 (3C, C = O), 164.36 (2C), 157.76 (C-OCH_3_), 144.33, 142.35 (C-S), 134.82, 129.49 (2C), 128.56, 128.43 (2C), 126.10 (2C), 126.07 (2C), 109.98, 104.73, 95.50 (2C), 57.91 (2C), 56.80 (O-CH_3_), 35.27 (2C), 35.00 (4C), 15.41 (S-CH_3_), 14.80 (C-CH_3_). Anal. Calcd. for C_34_H_35_ClN_2_O_9_S(%).C, 57.10; H, 4.93; N, 3.92 %. Found: C, 57.08; H, 4.89; N, 3.95%.

3-((2*S*,2′*R*)-7-chloro-4,6-dimethoxy-2′-methyl-3,4′,6′-trioxo-3H-spiro[benzofuran-2,1′-cyclohexan]-5′-yl)-3-(4-(methylthio)phenyl)-N-(2-oxo-2-((4-sulfamoylphenyl)amino)ethyl)propenamide **1j**.

Yield 83%, m.p. 245–247 °C.^1^H NMR (300 MHz, DMSO-*d*_6_) δ 10.04 (s, 1H, NH), 7.85–7.66 (m, 5H, 4CH+NH), 7.24–7.00 (m, 6H, 4CH+NH_2_), 6.36 (s, 1H, CH), 4.64 (t, *J* = 7.6 Hz, 1H, CH), 4.02 (s, 3H, OCH_3_), 3.88 (s, 3H, OCH_3_), 3.81 (d, *J* = 6.8 Hz, 2H, CH_2_), 3.07–2.86 (m, 2H, 2CH), 2.63–2.52 (m, 3H, 3CH), 2.41 (s, 3H, SCH_3_), 0.90 (d, *J* = 6.0 Hz, 3H, CH_3_). ^13^C NMR (75 MHz, DMSO-*d*_6_) δ 168.93 (3C, C = O), 164.37 (3C), 157.78 (C-OCH_3_), 138.74 (C-S-CH_3_), 128.48 (2C, C-S), 127.13 (3C), 126.20 (4C), 119.05 (3C), 91.18 (3C), 57.91 (2C), 56.84 (3C), 34.80 (3C), 15.48 (S-CH_3_), 14.80 (C-CH_3_). Anal. Calcd. for C_34_H_34_ClN_3_O_10_S_2_(%).C, 54.87; H, 4.60; N, 5.65%. Found: C, 54.89; H, 4.67; N, 5.55%.

#### 3.1.4. Synthesis of Usnic Acid Derivatives **4a**,**b**

The mixture of (*R*)-usnic acid **5** (0.52 g, 1.5 mmol) and corresponding amine (1.7 mmol) (0.2 g, 2.0 mmol of Et_3_N was added in the case of amine hydrochloride) was refluxed in EtOH (7 mL) for 1 h. Then obtained reaction mixture was cooled, diluted with 1% HCl solution (50 mL), stirred for 2 h at room temperature, and left overnight. The precipitate formed was collected by filtration, washed with 1% HCl solution (3*20 mL), H_2_O (3*20 mL), and dried to afford pure compounds **4a**,**b**.

(*R*)-4-((1-(6-acetyl-7,9-dihydroxy-8,9b-dimethyl-1,3-dioxo-3,9b-dihydrodibenzo[b,d]furan-2(1H)-ylidene)ethyl)amino)-3-hydroxybenzenesulfonamide **4a**.

Yield 67%, m.p. 201–203 °C.^1^H NMR (300 MHz, DMSO-*d*_6_) δ 14.68 (s, 1H, NH), 13.29 (s, 1H, OH), 11.86 (s, 1H, OH), 10.85 (br. s, 1H, OH), 7.61–7.10 (m, 5H, 3CH+NH_2_), 5.89 (s, 1H, CH), 2.68 (s, 3H, CH_3_), 2.59 (s, 3H, CH_3_), 2.03 (s, 3H, CH_3_), 1.77 (s, 3H, CH_3_). ^13^C NMR (75 MHz, DMSO-*d*_6_) δ 200.95 (C = O), 197.52 (C = O), 183.33 (C = O), 179.37, 169.23 (C-CH_3_), 162.74 (C-OH), 157.57 (C-OH), 156.65, 147.79, 137.26 (C-SO_2_), 131.57, 122.91, 122.75, 111.32, 108.72, 107.53, 103.76, 103.34, 102.57, 61.10 (C-CH_3_), 31.28 (CO-CH_3_), 28.47 (C-CH_3_), 18.54 (C-CH_3_), 18.15 (C-CH_3_). Anal. Calcd. for C_25_H_25_N_2_O_9_S(%).C, 56.70; H, 4.76; N, 5.29 %. Found: C, 56.69; H, 4.69; N, 5.30%.

(*R*)-3-((1-(6-acetyl-7,9-dihydroxy-8,9b-dimethyl-1,3-dioxo-3,9b-dihydrodibenzo[b,d]furan-2(1H)-ylidene)ethyl)amino)-4-hydroxybenzenesulfonamide **4b**.

Yield 54%, m.p. 215–217 °C.^1^H NMR (300 MHz, DMSO-*d*_6_) δ 14.64 (s, 1H, NH), 13.29 (s, 1H, OH), 11.86 (s, 1H, OH), 11.01 (br. s, 1H, OH), 7.69 (s, 2H, 2CH), 7.19–6.95 (m, 3H, CH+NH_2_), 5.88 (s, 1H, CH), 2.66 (s, 3H, CH_3_), 2.58 (s, 3H, CH_3_), 2.02 (s, 3H, CH_3_), 1.76 (s, 3H, CH_3_). ^13^C NMR (75 MHz, DMSO-*d*_6_) δ 201.35 (C = O), 198.60 (C = O), 174.93, 174.88, 163.00 (2C), 157.88 (C-OH), 156.08, 155.03, 135.46 (C-S), 127.65, 125.34 (2C), 123.41, 116.88 (2C), 106.95 (C-CH_3_), 105.46, 103.08, 57.20, 32.07 (CO-CH_3_), 31.51 (C-CH_3_), 20.85 (2C, C-CH_3_). Anal. Calcd. for C_24_H_22_N_2_O_9_S(%).C, 56.03; H, 4.31; N, 5.44%. Found: C, 56.01; H, 4.37; N, 5.38%.

### 3.2. CA Inhibition Assay

An applied photophysics stopped-flow instrument was used for assaying the CA-catalyzed CO_2_ hydration activity. Phenol red (at a concentration of 0.2 mM) was used as an indicator, working at the absorbance maximum of 557 nm, with 20 mM Hepes (pH 7.4) for α-class as a buffer, 20 mM TRIS (pH 8.3) for β- and γ-class as a buffer, and 20 mM Na_2_SO_4_ (for maintaining constant ionic strength), following the initial rates of the CA-catalyzed CO_2_ hydration reaction for a period of 10–100 s. The CO_2_ concentrations ranged from 1.7 to 17 mM for the determination of the kinetic parameters and inhibition constants. The non-catalyzed CO_2_ hydration was not subtracted from these curves and accounts for the remaining observed activity even at a high concentration of inhibitor, being in the range of 16–25%. However, the background activity from the uncatalyzed reaction is always subtracted when IC_50_ values are obtained by using the data analysis software for the stopped-flow instrument. Enzyme concentrations ranged between 5 and 10 nM. For each inhibitor, at least six traces of the initial 5–10% of the reaction were used for determining the initial velocity. The uncatalyzed rates were determined in the same manner and subtracted from the total observed rates. Stock solutions of the inhibitor (0.1 mM) were prepared in distilled–deionized water, and dilutions up to 0.01 nM were done thereafter with the assay buffer. Inhibitor and enzyme solutions were preincubated together for 15 min at room temperature prior to the assay to allow for the formation of the E–I complex. The inhibition constants were obtained by non-linear least-squares methods using PRISM 3 and the Cheng–Prusoff equation, as reported earlier, and represent the mean from at least three different determinations. All CA isoforms were recombinant proteins obtained in-house, as reported earlier [58,59,60] and their concentrations in the assay system were in the range of 6.2–14.8 nM.

### 3.3. Molecular Docking Studies

Molecular modeling studies were performed using the software AutoDock 4.2 (The Scripps Research Institute, La Jolla, CA, USA) [61]. Protein Data Bank was also used in order to obtain the he crystal structures ofhCA I (PDB code 3W6H) and *h*CA II (PDB code 3HS4) cytosolic isoforms as well as *h*CA IX (PDB code 3IAI) transmembrane tumor-associated isoform [62]. All the procedure was carried out as in our previous works [41,42].

### 3.4. Drug Likeness

The study was performed as described in our previous paper [41].

## 4. Conclusions

In conclusion, we synthetized and investigated a novel griseofulvin and usnic acid sulfonamides for their effective inhibition against three relevant human carbonic anhydrase isoforms, such as the ubiquitous hCA I and hCA II isoforms and the tumor-associated isoforms hCA IX, which are involved in many diseases such as glaucoma, retinitis pigmentosa, epilepsy, and tumors. Furthermore, the inhibitory activity against β- and γ-CAs from three different bacterial and one fungal strain was evaluated. Six compounds (**1b**–**1d**, **1h**, **1i** and **1j**) were more potent than AAZ against hCA I while five (**1d**, **1h**, **1i**, **1j** and **4a**) showed better activity than AAZ against the hCA II isoform. It should be mentioned that compound **1d** demonstrated the best activity with Ki 4.9 nm and selectivity index (SI) towards hCA I and hCA IX isoforms 5.88 and 6.00 respectively. The investigation of CA inhibitory activity against bacterial stains (*Burkholderia pseudomallei-BpsCAγ*, *Porphyromonas gingivalis* (*PgiCAβ* and *PgiCAγ*), *Colwellia psychrerythraea* (CpsCA) as well as *Streptococcus mutans*, *Malassezia globosa*) revealed *that* only in case of *Pgi*CA*γ, Csp*CA*γ* and *St. mutans* some compounds excited the activity of reference drug Moreover, all compounds appeared to be very potent against the MgCA with a Ki lower than that of the reference drug. The comparison of activity towards MgCA with hCA isoforms revealed that among the most active compounds, only one is common (**1d**. Furthermore, computational procedures were used to investigate the binding mode of this class of compounds against hCA isoforms, and the obtained results were in agreement with the experimental data.

## Figures and Tables

**Table 1:** Structures of compounds.

No	Structure	No	Structure
**1a**	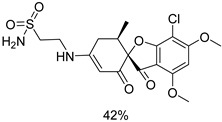	**1g**	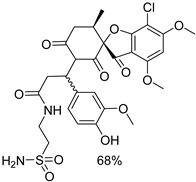
**1b**	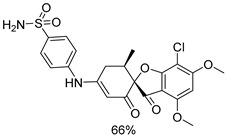	**1h**	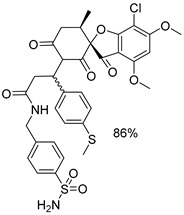
**1c**	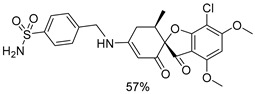	**1i**	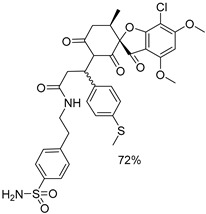
**1d**	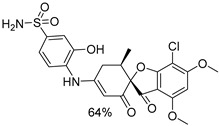	**1j**	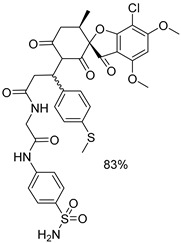
**1e**	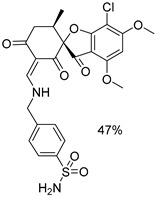	**4a**	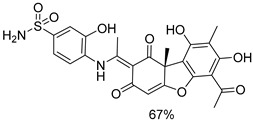
**1f**	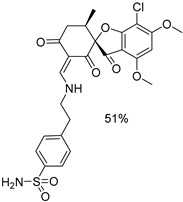	**4b**	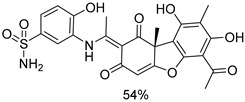

**Table 2:** Inhibition data of human CA isoforms I, II, IX, and β, γ from some bacterial and fungal strains with titled compounds and AAZ by a stopped-flow CO_2_ hydrase assay.

K_I_ (nM) *									
Cmp	hCA I	hCA II	hCAIX	MgCA	PgiCAβ	PgiCAγ	SmuCA	BpsCAγ	CpsCAγ
**1a**	562.0	653.4	31.2	544.0	778.6	92.1	1629	448.2	3149
**1b**	233.6	64.4	203.1	475.6	818.9	820.3	293.7	945.0	938.9
**1c**	74.6	29.1	102.0	568.5	896.2	660.6	3997	833.0	899.9
**1d**	28.8	4.9	29.4	1589	1285	584.2	897.0	877.5	591.4
**1e**	320.2	165.1	29.2	1826	939.6	1273	1638	1061	219.6
**1f**	347.6	70.8	30.6	4000	8361	601.5	2468	2282	726.3
**1g**	2986	4052	709.6	3148	5593	2219	1218	2334	301.0
**1h**	41.1	7.5	73.7	4346	5754	853.4	579.8	1401	625.0
**1i**	15.3	8.1	23.3	1912	506.2	853.9	889.4	1038	804.4
**1j**	27.7	5.7	22.5	2047	2998	2320	1234	710.5	176.8
**4a**	369.5	8.4	66.9	3430	888.2	896.7	841.3	472.9	723.2
**4b**	1692	755.3	305.0	2497	766.3	1841	1696	1000	809.3
**AAZ**	250.0	12.8	25.6	40,000	214	324	344	149	502

* Mean from 3 different assays, by a stopped flow technique (errors were in the range of ±5–10% of the reported values).

**Table 3:** Molecular docking free binding energies (kcal/mol) and interactions of tested compounds on hCA I, II and IX isoforms.

No	*h*CA Isoform	Estimated Free Binding Energy (Kcal/mol)	Chelating the Zn (II) Ion	Residues Involved in H-Bond Interactions	Residues Involved in Hydrophobic Interactions
**1d**	hCA I	−10.35	Yes	Thr199	Ala121,Leu198, Thr199, His200
	hCA II	−12.02	Yes	Thr199, Thr200	Ile91,Val121, Phe131, Leu198, Trp209
	hCA IX	−8.53	Yes	Thr199	Val121, Val131, Leu198
**1i**	hCA I	−12.45	Yes	Thr199	Trp5, Ile60, Val62, His64, Leu198
	hCA II	−11.64	Yes	Thr199	Val121, Leu198
	hCA IX	−12.74	Yes	Glu67, Gln92, Thr199, His199	Leu198, Thr200
**4a**	hCA I	−6.72	No	-	Ala121, Leu198
	hCA II	−10.23	Yes	Thr199	Ile91, Val121, Leu198, Thr200
	hCA IX	−6.57	No	-	Val121, Leu198, Trp209
**4b**	hCA I	−5.11	No	-	Leu198
	hCA II	−4.89	No	-	Val121, Phe131, Leu198
	hCA IX	−6.26	No	-	Val121, Leu198
**AAZ**	hCA I	−8.28	Yes	Gln92	Leu198, Thr199, His200, Pro201, Trp209
	hCA II	−8.87	Yes	Thr199, Thr200	Val121, Phe131, Leu198, Trp209
	hCA IX	−9.02	Yes	Thr199, Thr200	Val121, Val143, Val131, Leu198, Trp209

**Table 4:** Drug likeness predictions of tested compounds.

No	MW	Number of HBA ^a^	Number of HBD ^b^	Log *P*_o/w_ (iLOGP) ^c^	Log S ^d^	TPSA ^e^	Lipinski	Bioavailability Score	Drug-Likeness Model Score
**1a**	444.89	8	2	1.19	Moderately soluble	142.40	0	0.55	0.70
**1b**	492.93	8	2	2.56	Moderately soluble	142.40	0	0.55	0.58
**1c**	506.96	8	2	2.36	Moderately soluble	142.40	1 violation: MW > 500	0.55	0.75
**1d**	508.93	9	3	2.33	Moderately soluble	162.63	1 violation: MW > 500	0.55	0.42
**1e**	534.97	9	2	2.78	Moderately soluble	159.47	1 violation: MW > 500	0.55	0.45
**1f**	548.99	9	2	2.68	Moderately soluble	159.47	1 violation: MW > 500	0.55	0.47
**1g**	639.07	12	3	192	Poorly Soluble	206.00	2 violations: MW > 500, NorO > 10	0.17	1.24
**1h**	701.21	10	2	3.58	Poorly soluble	201.84	2 violations: MW > 500, NorO > 10	0.17	1.19
**1i**	715.23	9	2	3.40	Poorly soluble	201.84	1 violation: MW > 500	0.55	1.13
**1j**	744.23	11	3	3.44	Poorly soluble	230.94	2 violations: MW > 500, NorO > 10	0.17	1.12
**4a**	514.50	10	5	1.10	Moderately soluble	201.70	2 violations: MW > 500, NorO > 10	0.17	0.20
**4b**	514.50	10	5	1.15	Moderately soluble	201.70	2 violations: MW > 500, NorO > 10	0.17	0.13

^a^ number of hydrogen bond acceptors; ^b^ number of hydrogen bond donors; ^c^ lipophilicity; ^d^ Water solubility (SILICOS-IT [S = Soluble]); ^e^ topological polar surface area (Å^2^).

## Data Availability

Not applicable.

## Supplementary Materials

The following supporting information can be downloaded at: https://www.mdpi.com/article/10.3390/ijms24032802/s1. References [61,62,63] are cited in the supplementary materials.
