# Supplementary material for: Synthesis, Biological and In Silico Studies of Griseofulvin and Usnic Acid Sulfonamide Derivatives as Fungal, Bacterial and Human Carbonic Anhydrase Inhibitors
Source: Int J Mol Sci. 2023 Feb 1;24(3):2802. doi: 10.3390/ijms24032802 (PMC9917406; doi:10.3390/ijms24032802)
Supplement: Supplementary file 1 [file ijms-24-02802-s001.zip › ijms-2141157-supplementary.pdf]

Synthesis, Biological and In Silico Studies of Griseofulvin and usnic acid sulfonamide derivatives as Carbonic Anhydrase Inhibitors

Andrea Angeli [1,2], Victor Kartsev [3], Anthi Petrou [4], Boris Lichitsky [5], Andrey Komogortsev [5], Clemente Capasso [2], Athina Geronikaki [4,*] and Claudiu T. Supuran [1,*]

[1] NeuroFarba Department, Sezione di ScienzeFarmaceutiche, Università degli Studi di Firenze, Via Ugo Schiff 6, 50019 Sesto Fiorentino, Italy

[2] Istituto di Bioscienze e Biorisorse, CNR, Via Pietro Castellino 111, 80131 Napoli, Italy.

[3] InterBioScreen, 142432 Chernogolovka, Russia

[4] Department of Pharmacy, School of Health, Aristotle University of Thessaloniki, 54124 Thessaloniki, Greece;

[5] Zelinsky Institute of Organic Chemistry, Leninsky Prospect, 119991 Moscow, Russia;

* Correspondence: geronik@pharm.auth.gr; claudiu.supuran@unifi.it

^{1}H-NMR and ^{13}C-NMR of compounds

Compound 1a

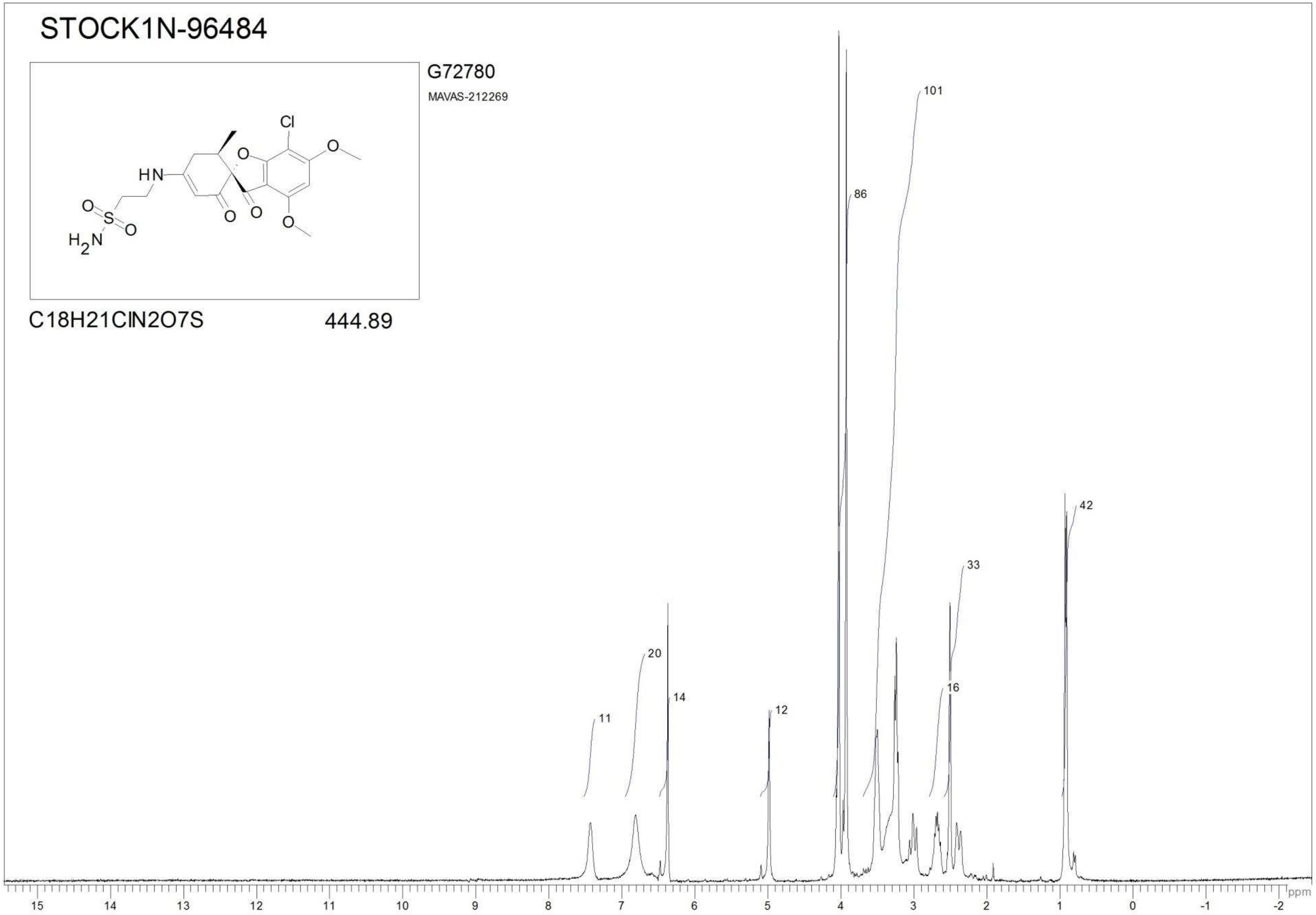

STOCK1N-96484
G72780
MAVAS-212269
Cl
O
HN
O
S
O
H2N
O
O
O
O
C18H21ClN2O7S
444.89
101
86
42
33
20
16
14
12
11
ppm
15
14
13
12
11
10
9
8
7
6
5
4
3
2
1
0
-1
-2

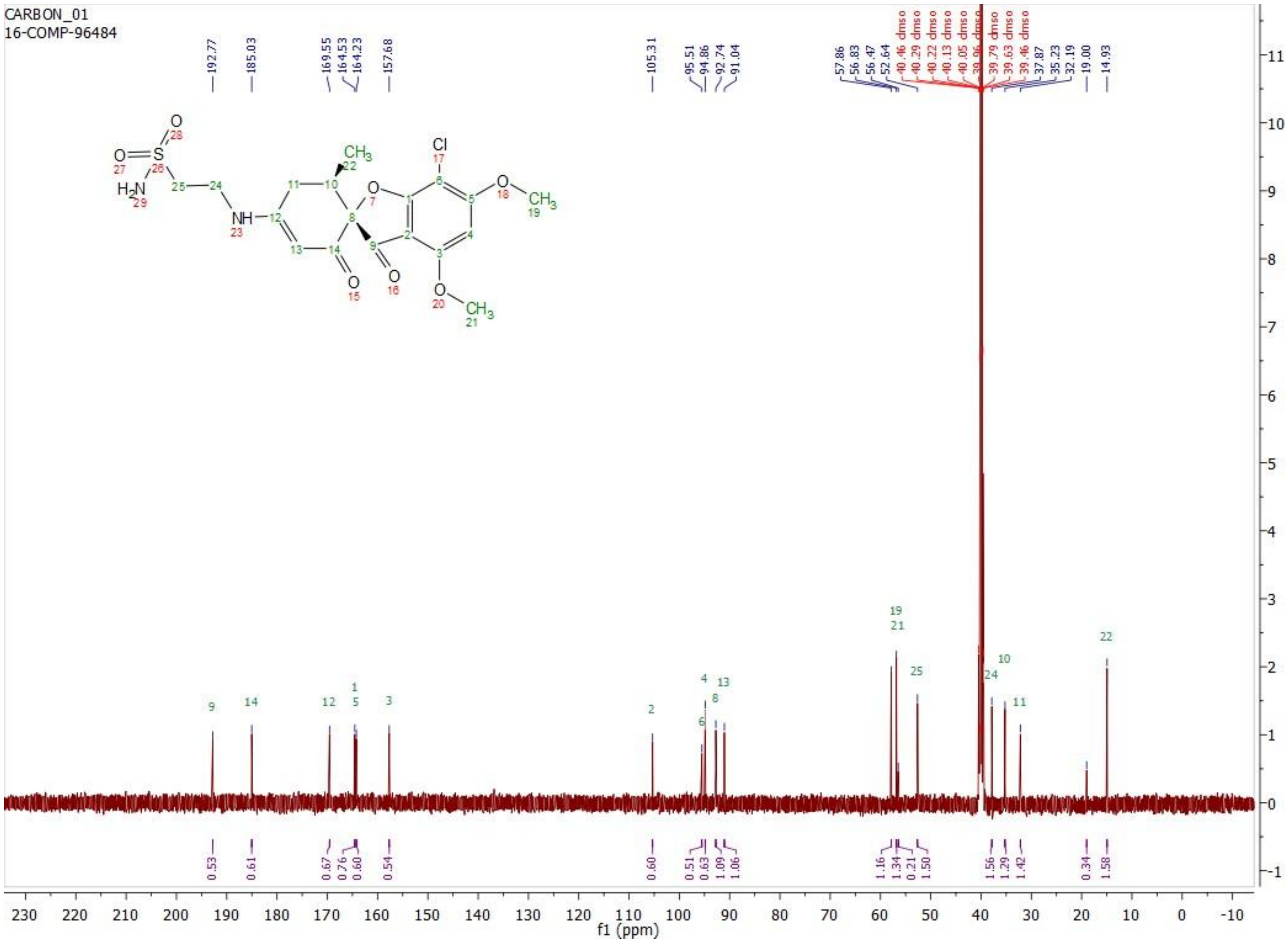
CARBON_01
16-COMP-96484
192.77
185.03
169.55
164.53
164.23
157.68
105.31
95.51
94.86
92.74
91.04
57.86
56.83
56.47
52.64
40.46 dmso
40.29 dmso
40.22 dmso
40.13 dmso
40.05 dmso
39.96 dmso
39.79 dmso
39.63 dmso
39.46 dmso
37.87
35.23
32.19
19.00
14.93
0.53
0.61
0.67
0.76
0.60
0.54
0.60
0.51
0.63
1.09
1.06
1.16
1.34
0.21
1.50
1.56
1.29
1.42
0.34
1.58
f1 (ppm)

Compound 1b

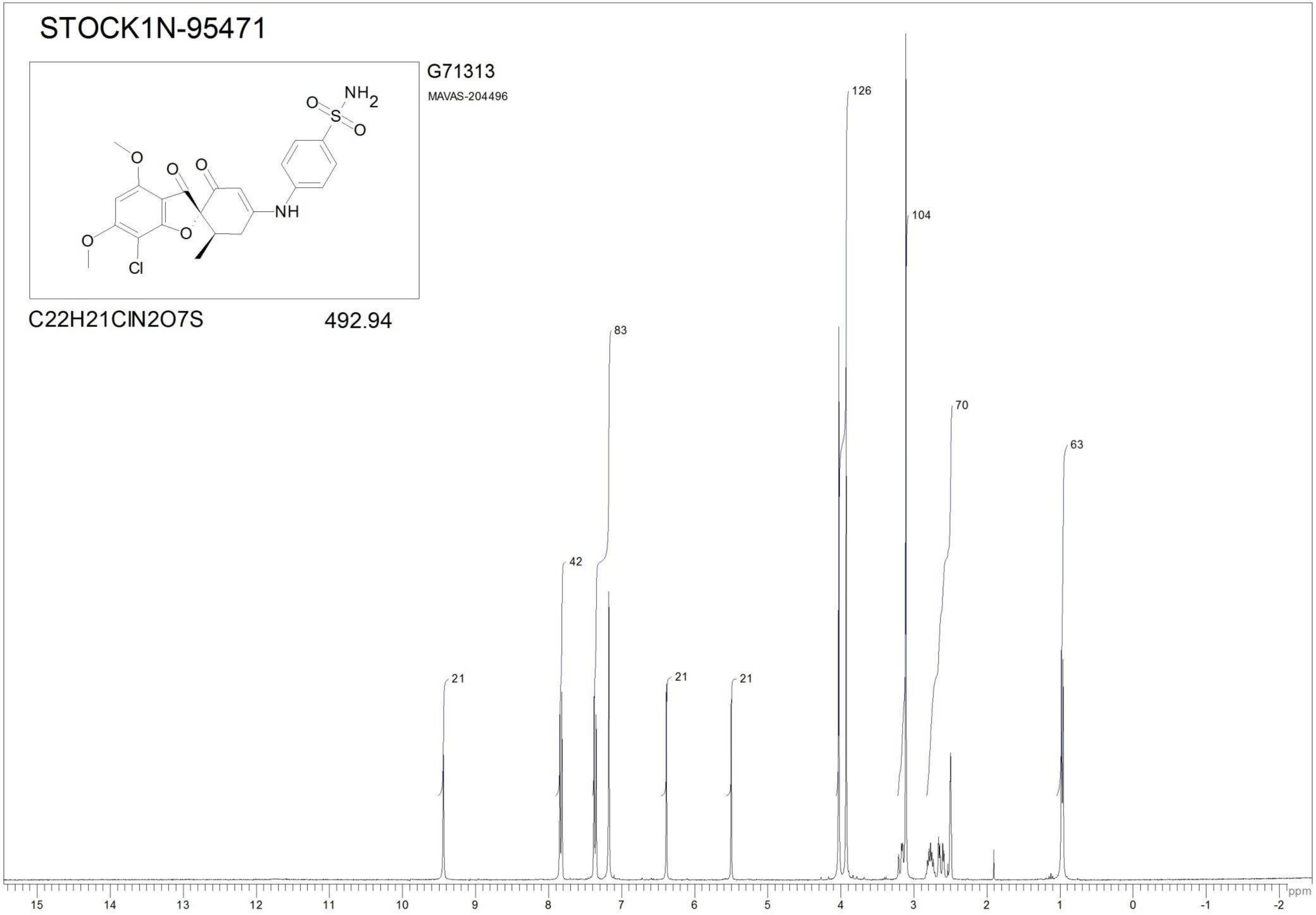
STOCK1N-95471
G71313
MAVAS-204496
C22H21ClN2O7S
492.94
21
42
83
21
21
126
104
70
63
ppm
15
14
13
12
11
10
9
8
7
6
5
4
3
2
1
0
-1
-2

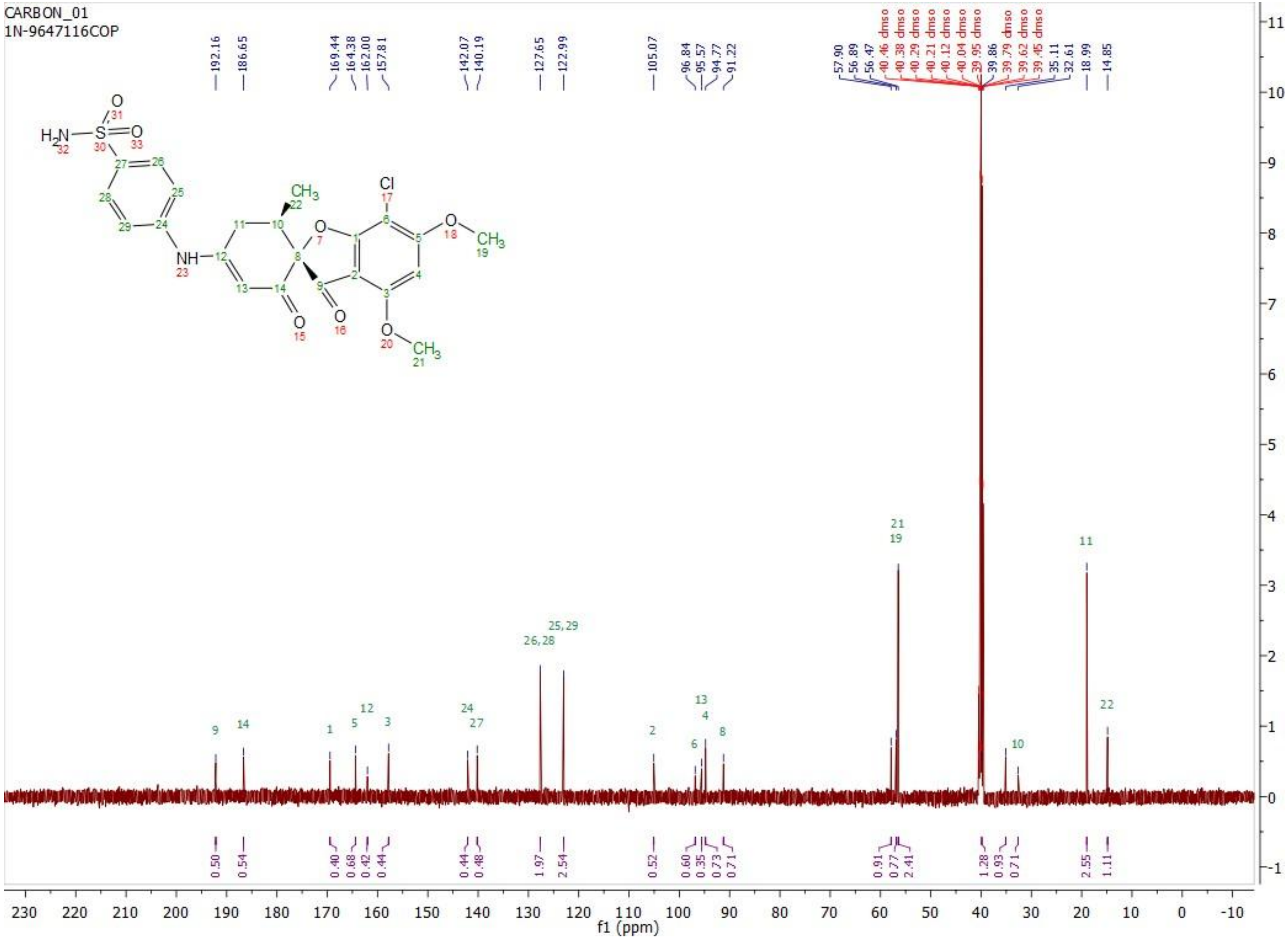

CARBON_01
1N-9647116COP
192.16
186.65
169.44
164.38
162.00
157.81
142.07
140.19
127.65
122.99
105.07
96.84
95.57
94.77
91.22
57.90
56.89
56.47
40.46 dmso
40.38 dmso
40.29 dmso
40.21 dmso
40.12 dmso
40.04 dmso
39.95 dmso
39.86
39.79 dmso
39.62 dmso
39.45 dmso
35.11
32.61
18.99
14.85
H_2N
S
O
Cl
O
CH_3
NH
f1 (ppm)

Compound 1c

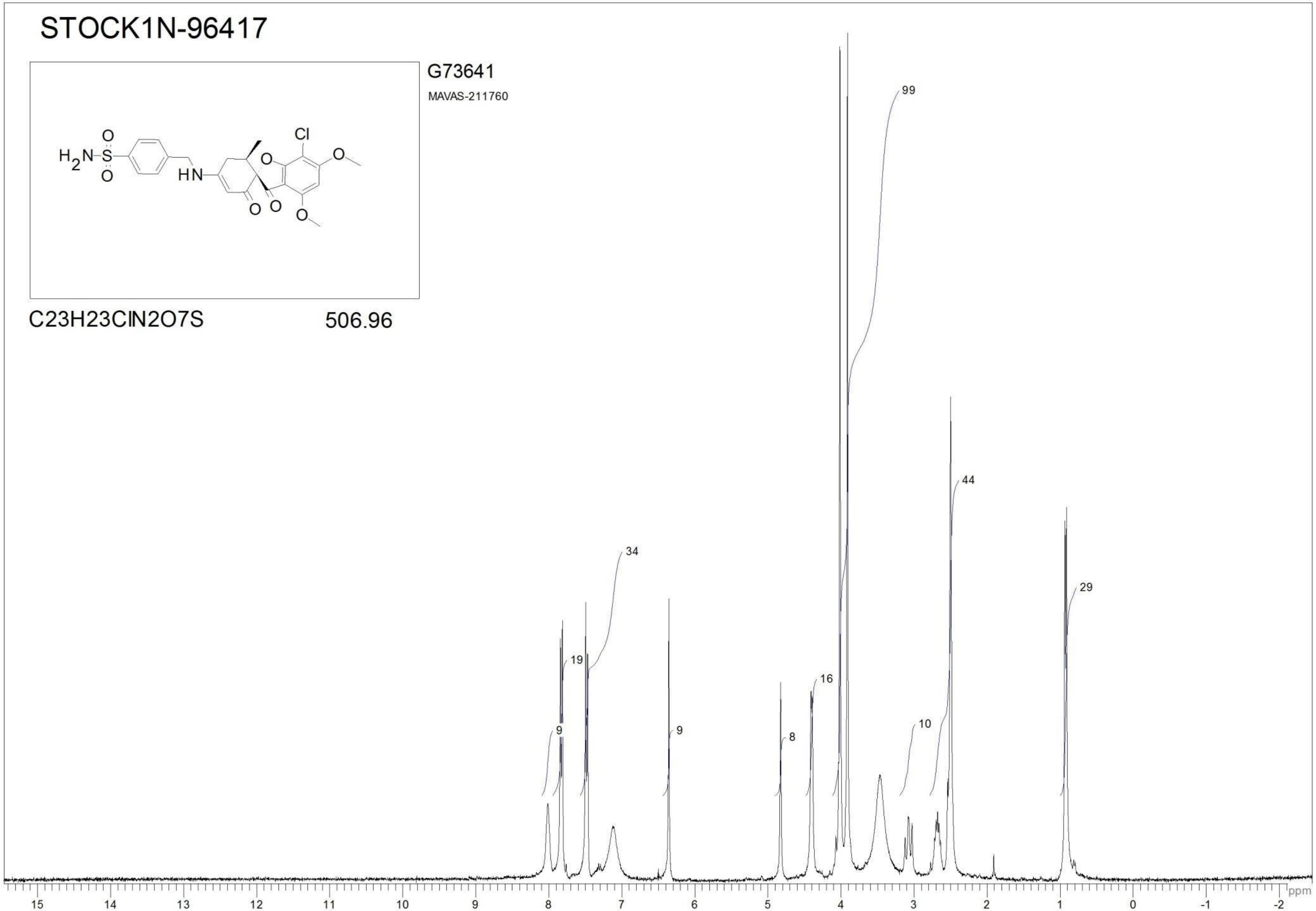
STOCK1N-96417
G73641
MAVAS-211760
H2N
S
O
O
HN
O
O
O
Cl
O
O
O
C23H23ClN2O7S
506.96
99
44
34
29
19
16
10
9
9
8
15
14
13
12
11
10
9
8
7
6
5
4
3
2
1
0
-1
-2
ppm

CARBON_01
1N-9647116COP

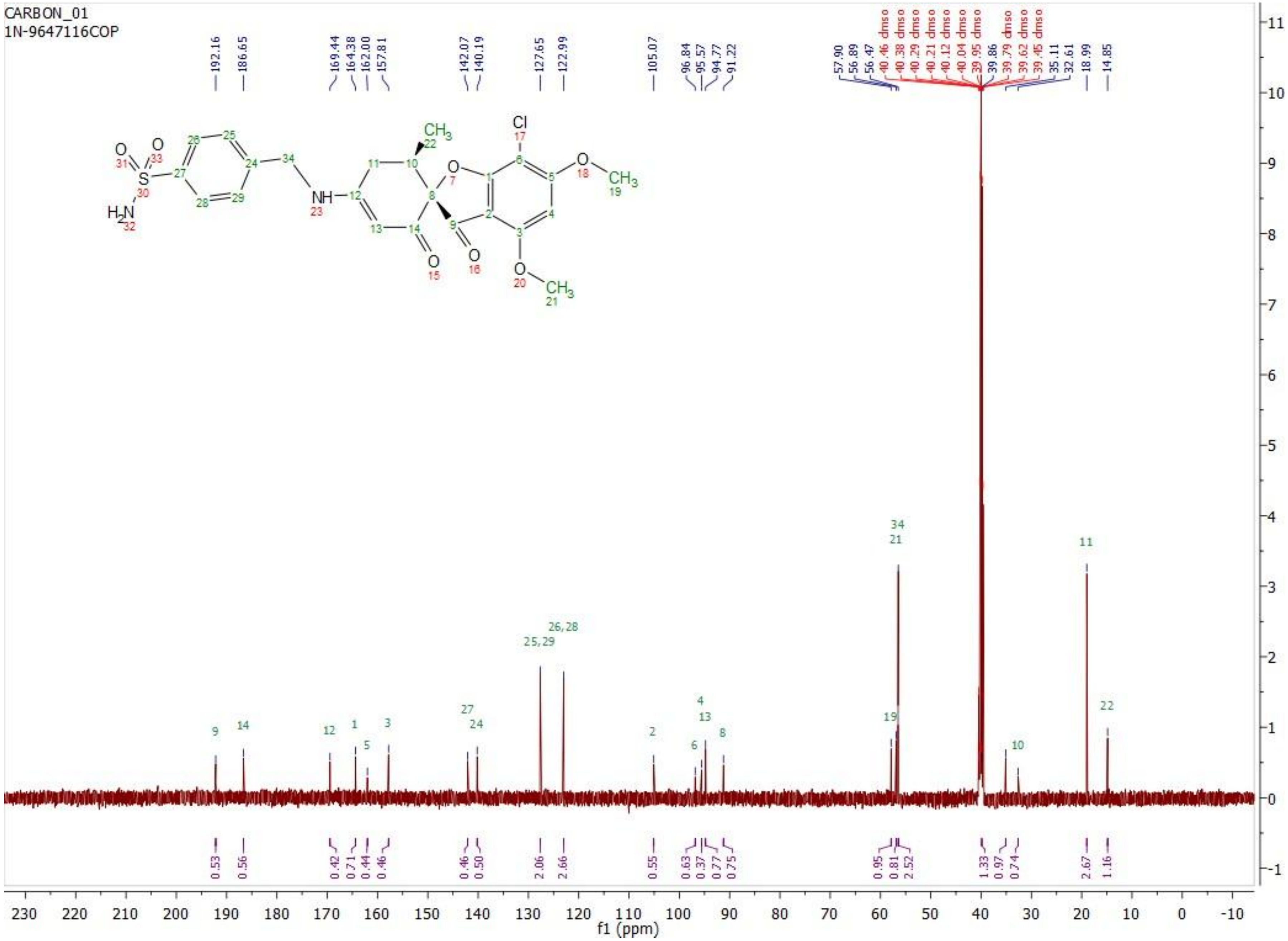

Compound 1d

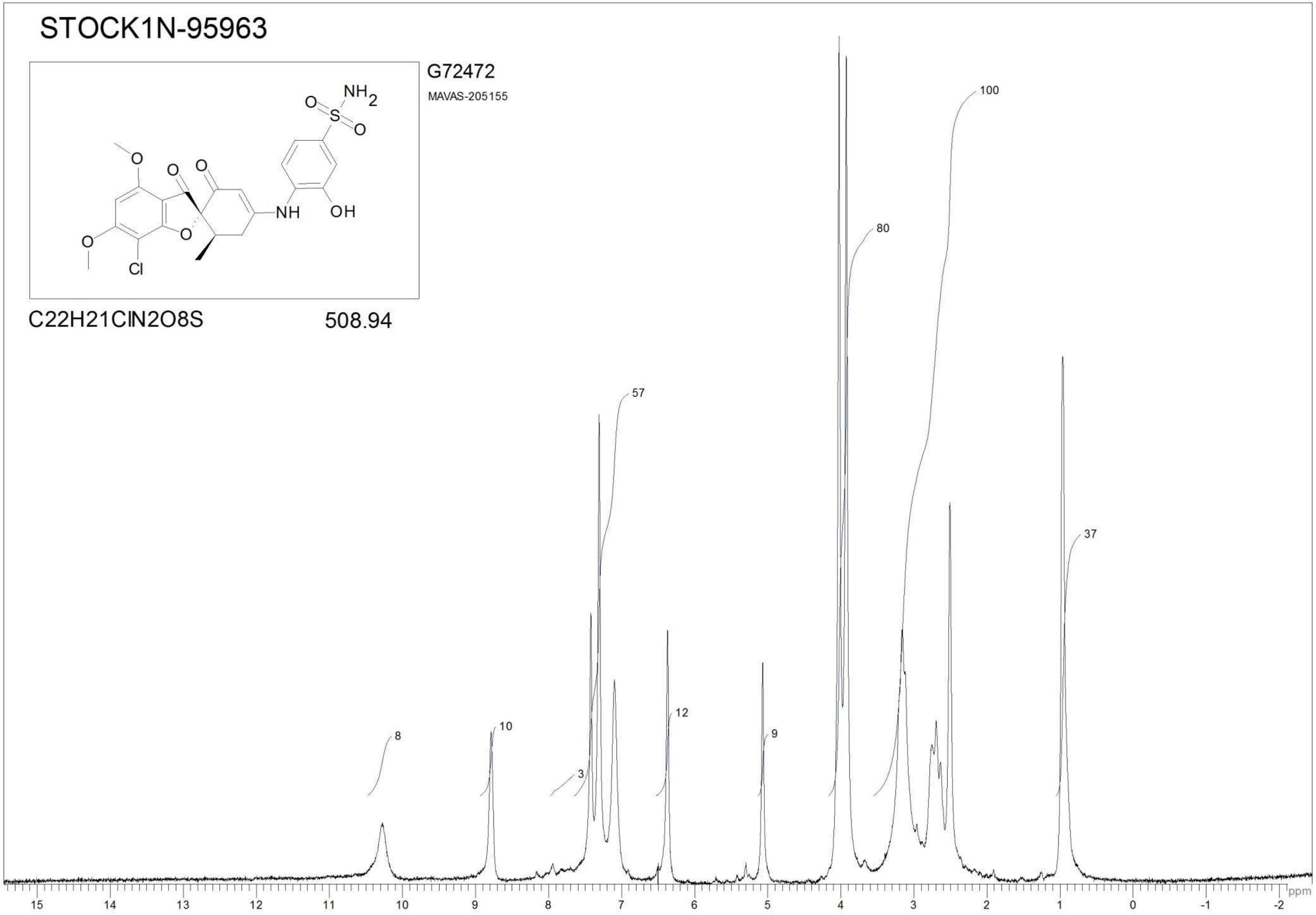

STOCK1N-95963
G72472
MAVAS-205155
C22H21ClN2O8S
508.94
O
NH2
S
O
O
O
O
NH
OH
O
O
Cl
100
80
57
37
8
10
3
12
9
ppm

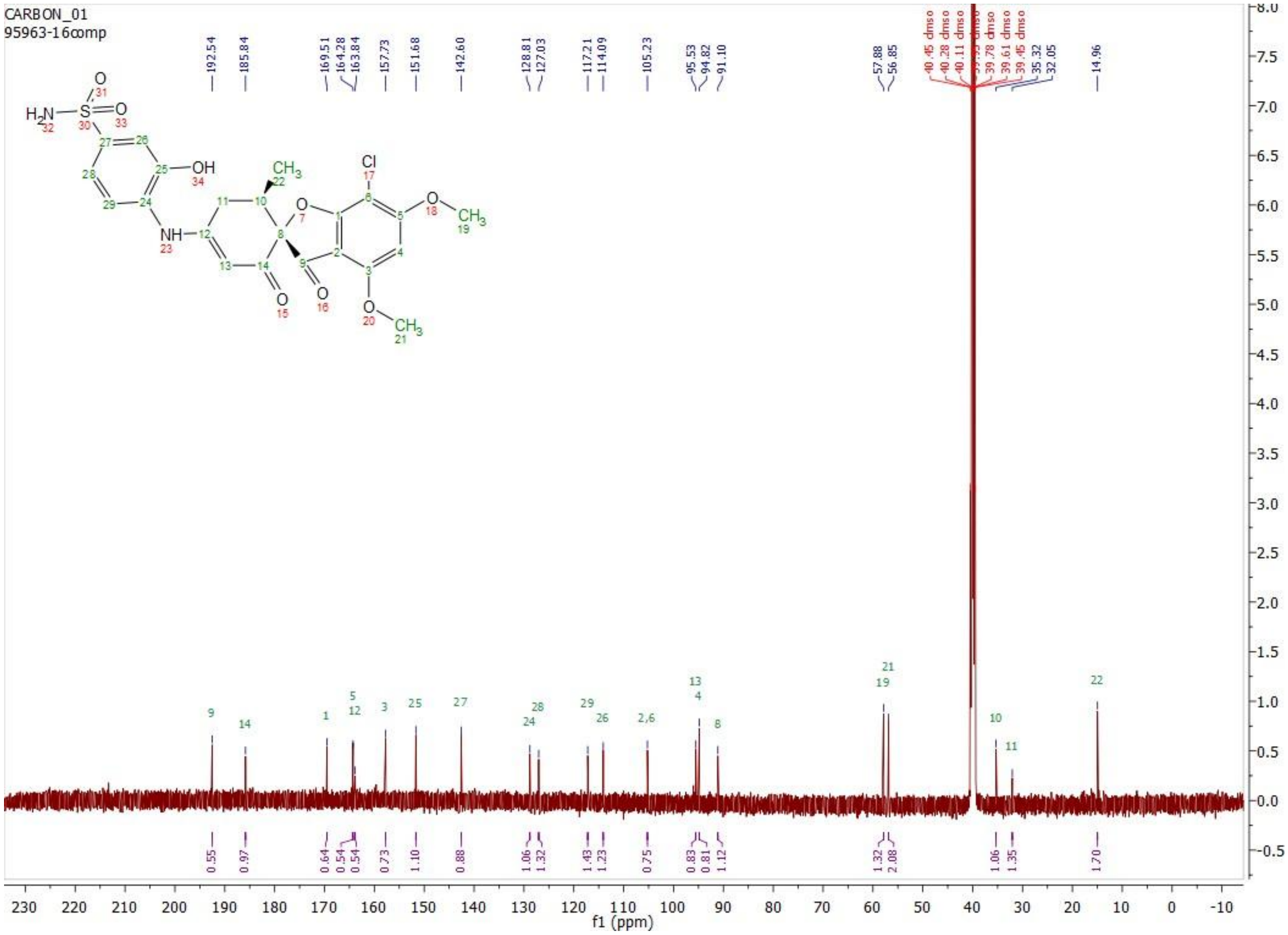
CARBON_01
95963-16comp
192.54
185.84
169.51
164.28
163.84
157.73
151.68
142.60
128.81
127.03
117.21
114.09
105.23
95.53
94.82
91.10
57.88
56.85
35.32
32.05
14.96
f1 (ppm)

Compound 1e

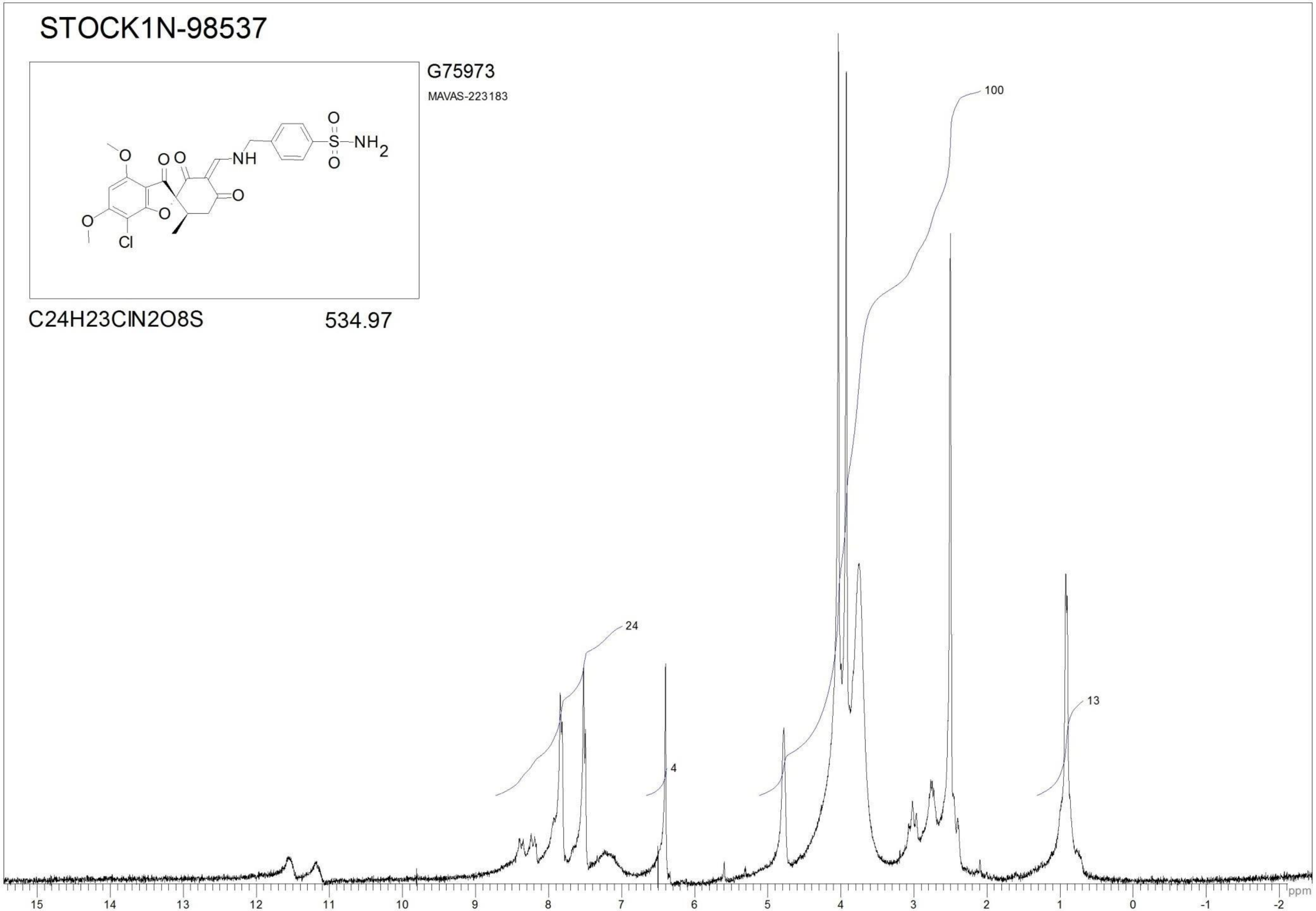
STOCK1N-98537
G75973
MAVAS-223183
O
NH
S
NH2
O
O
O
O
O
O
Cl
C24H23ClN2O8S
534.97
100
24
4
13
15
14
13
12
11
10
9
8
7
6
5
4
3
2
1
0
-1
-2
ppm

CARBON_01
in-98537

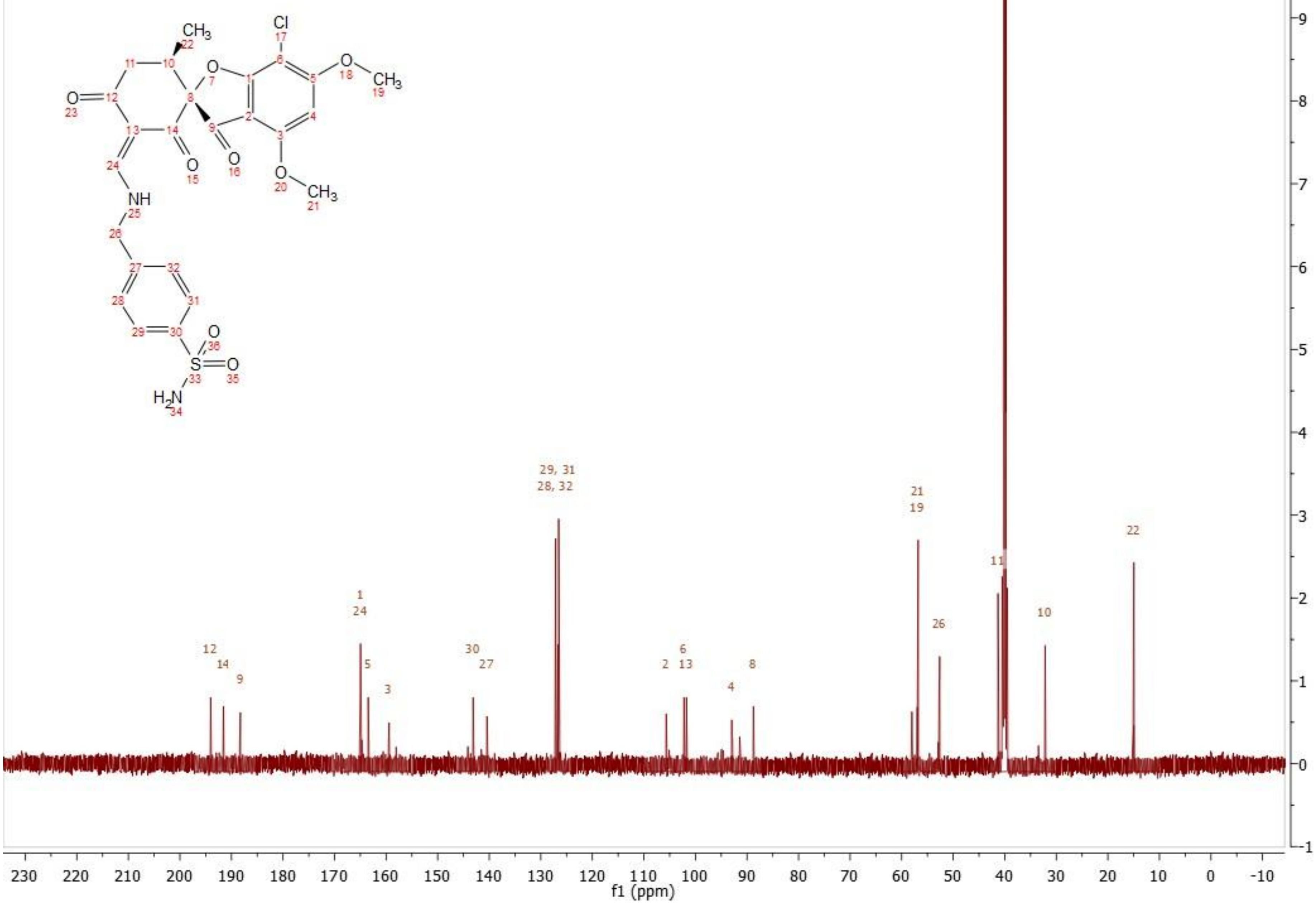

Compound 1f

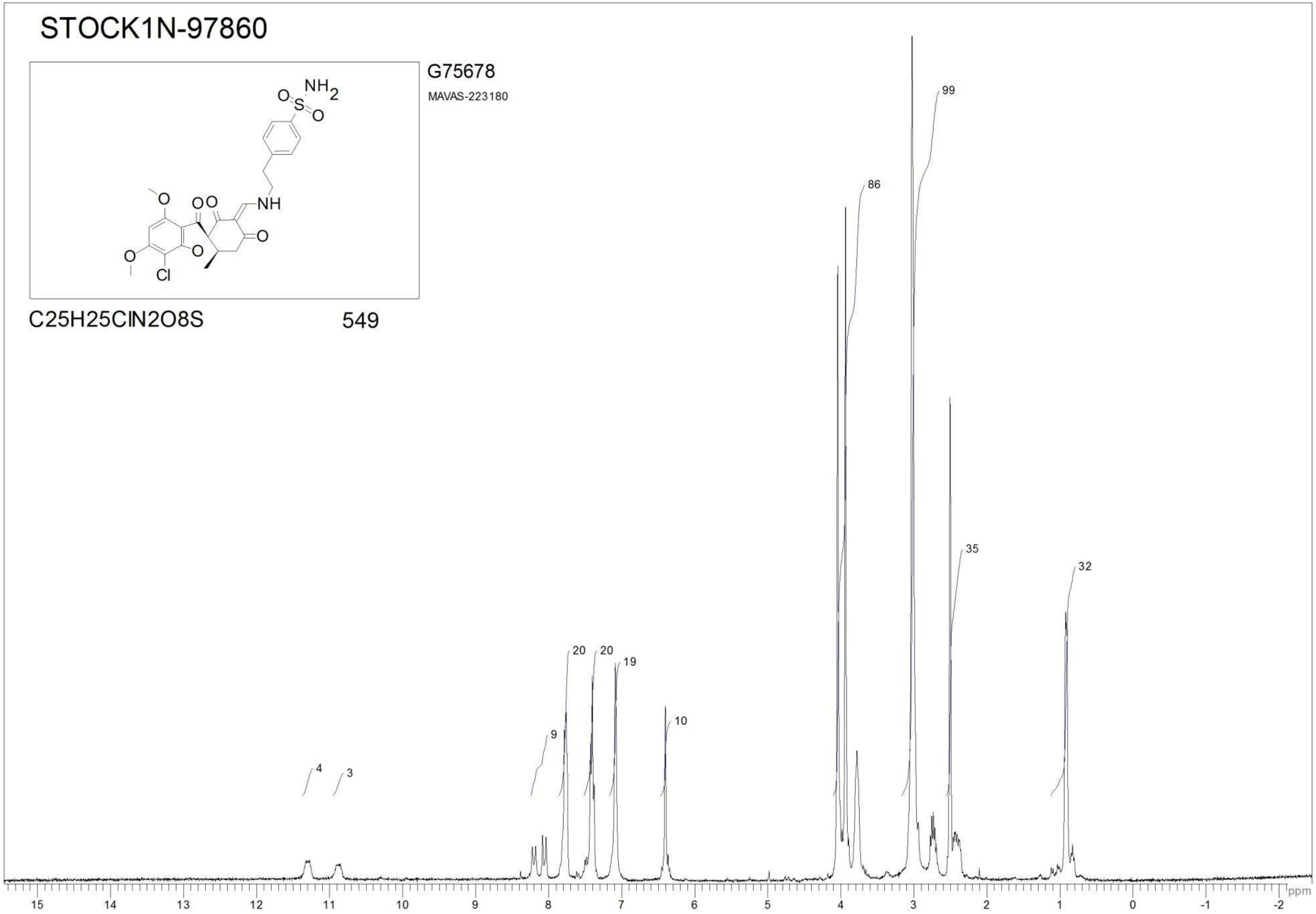
STOCK1N-97860
G75678
MAVAS-223180
C25H25ClN2O8S
549
4
3
9
20
20
19
10
86
99
35
32
ppm
15
14
13
12
11
10
9
8
7
6
5
4
3
2
1
0
-1
-2

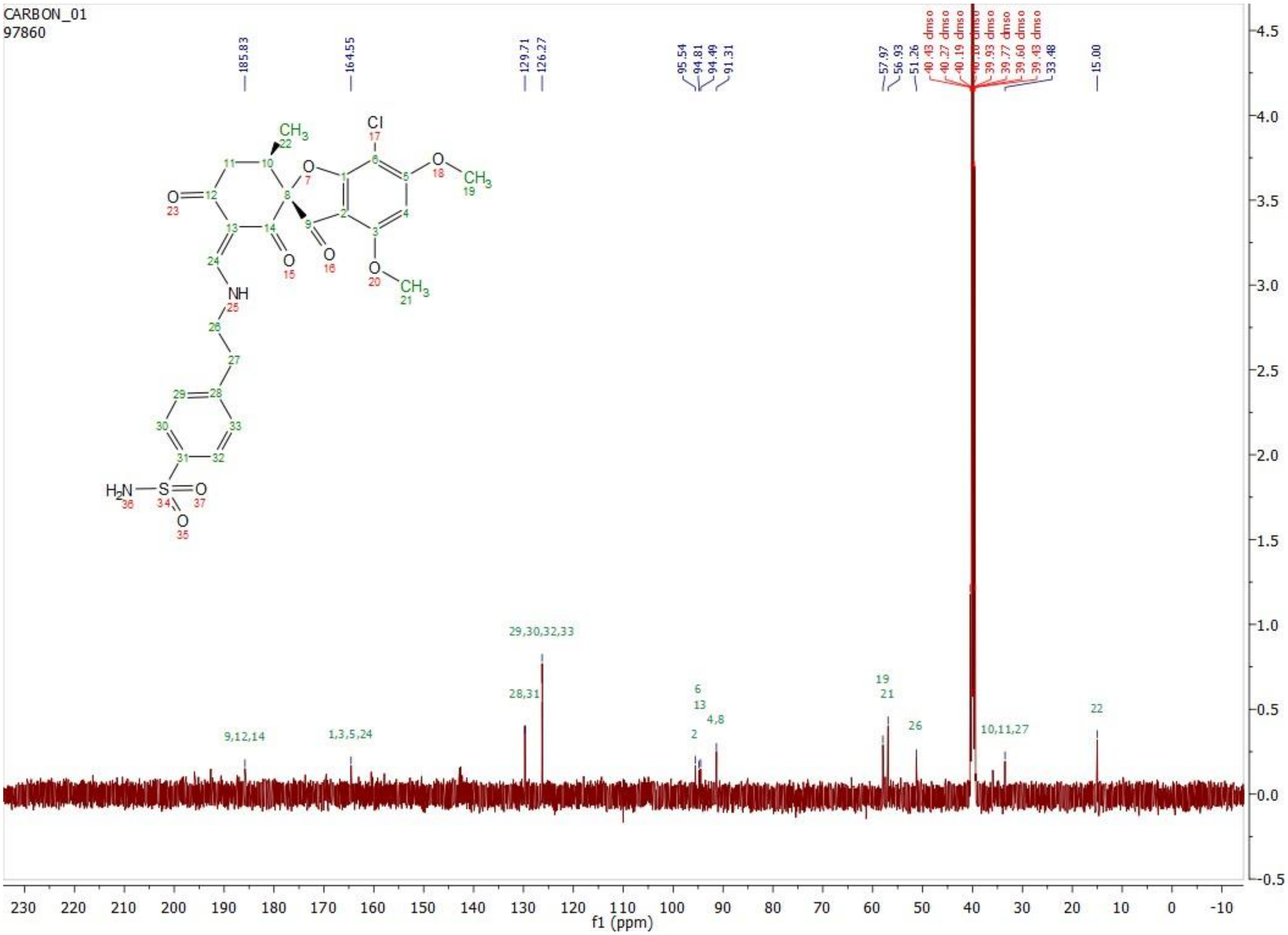

CARBON_01
97860
185.83
164.55
129.71
126.27
95.54
94.81
94.49
91.31
57.97
56.93
51.26
40.43 dmso
40.27 dmso
40.19 dmso
39.93 dmso
39.77 dmso
39.60 dmso
39.43 dmso
33.48
15.00
9,12,14
1,3,5,24
29,30,32,33
28,31
6
13
4,8
2
19
21
26
10,11,27
22
f1 (ppm)

Compound 1g

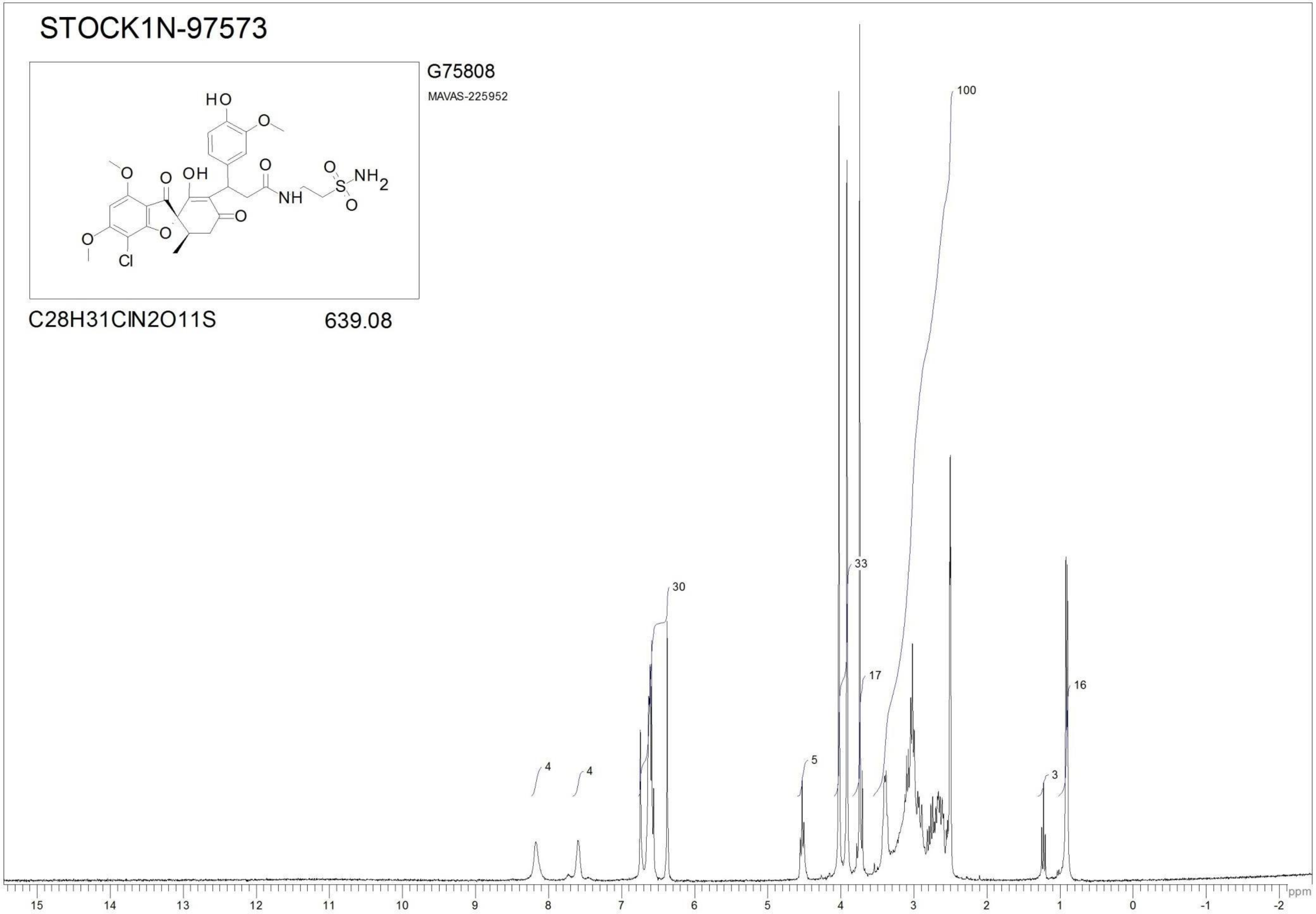
STOCK1N-97573
G75808
MAVAS-225952
HO
O
OH
O
O
NH
NH2
O
O
Cl
C28H31ClN2O11S
639.08
100
33
30
17
16
5
4
4
3
15
14
13
12
11
10
9
8
7
6
5
4
3
2
1
0
-1
-2
ppm

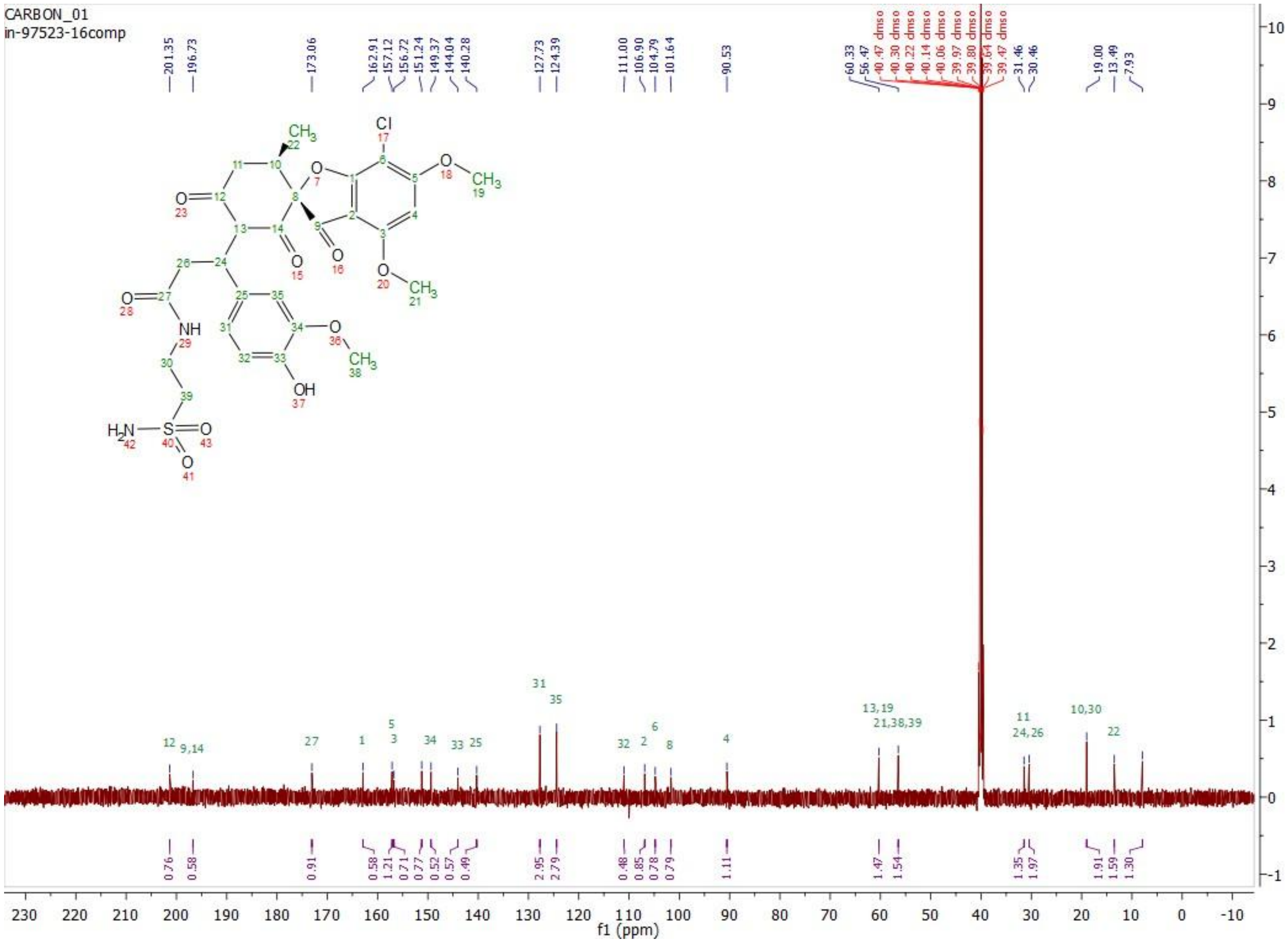
CARBON_01
in-97523-16comp
201.35
196.73
173.06
162.91
157.12
156.72
151.24
149.37
144.04
140.28
127.73
124.39
111.00
106.90
104.79
101.64
90.53
60.33
56.47
40.47 dmso
40.30 dmso
40.22 dmso
40.14 dmso
40.06 dmso
39.97 dmso
39.80 dmso
39.64 dmso
39.47 dmso
31.46
30.46
19.00
13.49
7.93
12
9,14
27
1
5
3
34
33 25
31
35
32 2
6
8
4
13,19
21,38,39
11
24,26
10,30
22
0.76
0.58
0.91
0.58
1.21
0.71
0.77
0.52
0.57
0.49
2.95
2.79
0.48
0.85
0.78
0.79
1.11
1.47
1.54
1.35
1.97
1.91
1.59
1.30
f1 (ppm)

Compound 1h

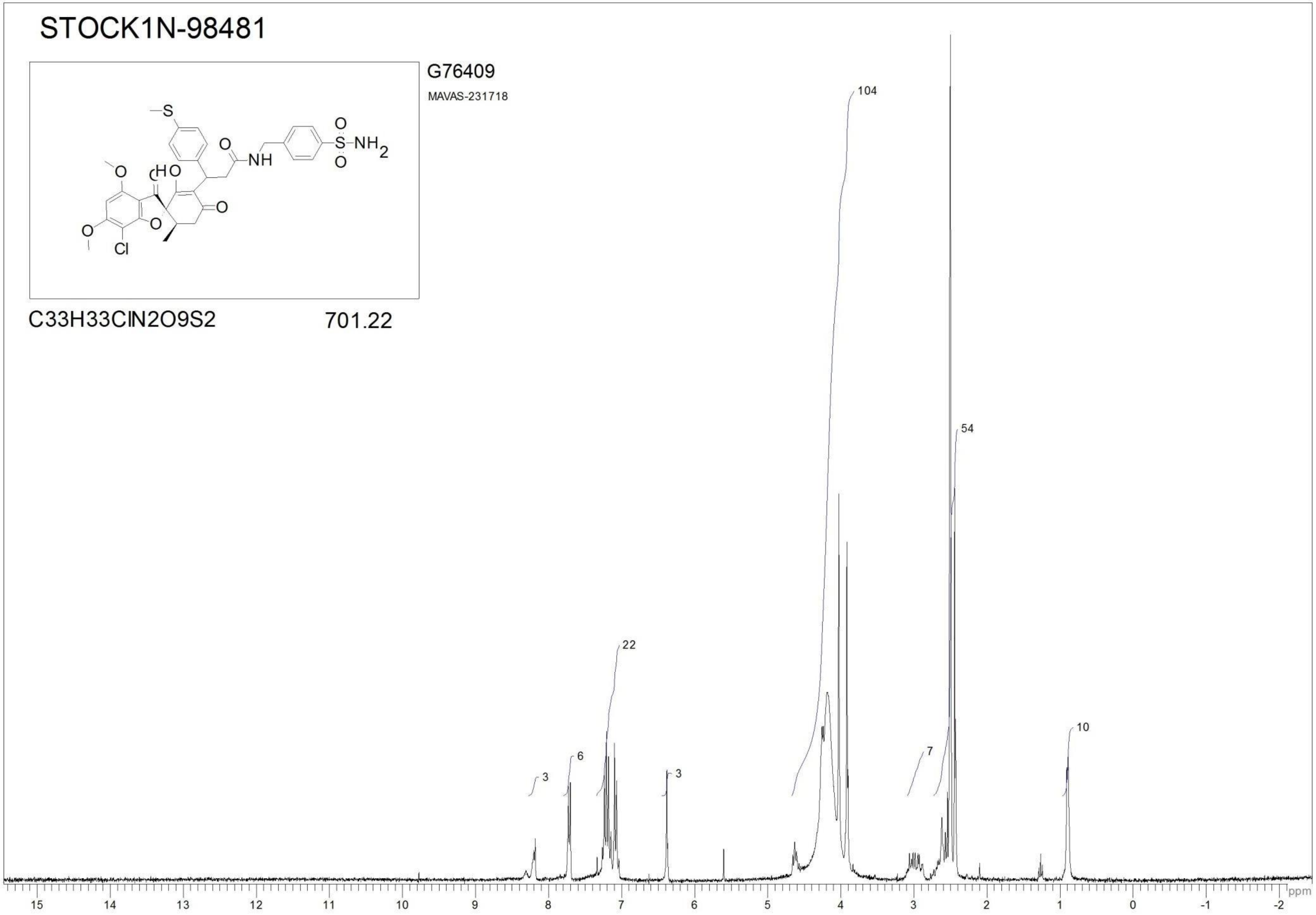
STOCK1N-98481
G76409
MAVAS-231718
S
O
NH
S
NH2
O
O
HO
O
O
O
O
O
Cl
C33H33ClN2O9S2
701.22
104
54
22
10
7
6
3
3
15
14
13
12
11
10
9
8
7
6
5
4
3
2
1
0
-1
-2
ppm

CARBON_01
1n-98481-16comp

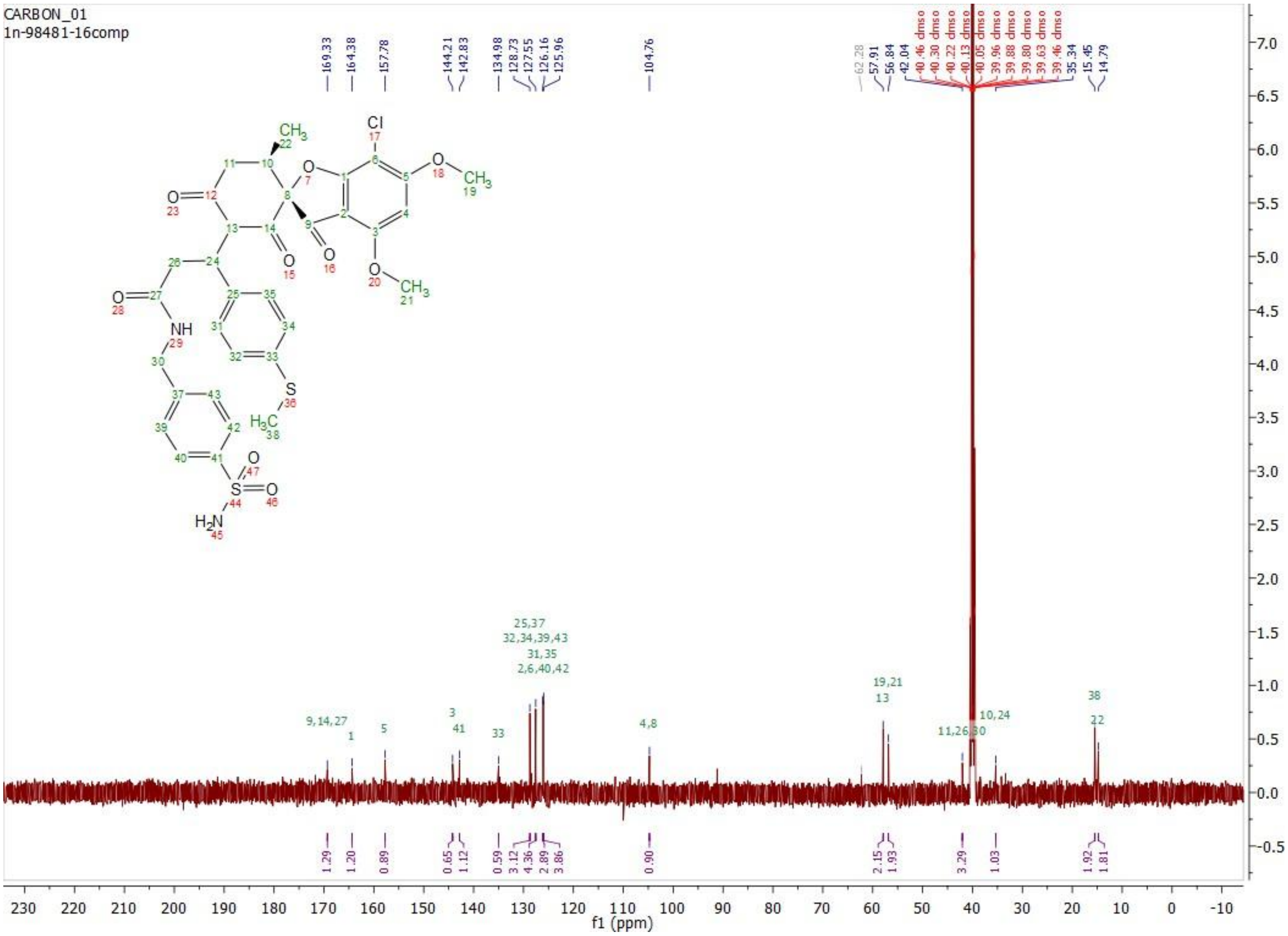

Compound 1i

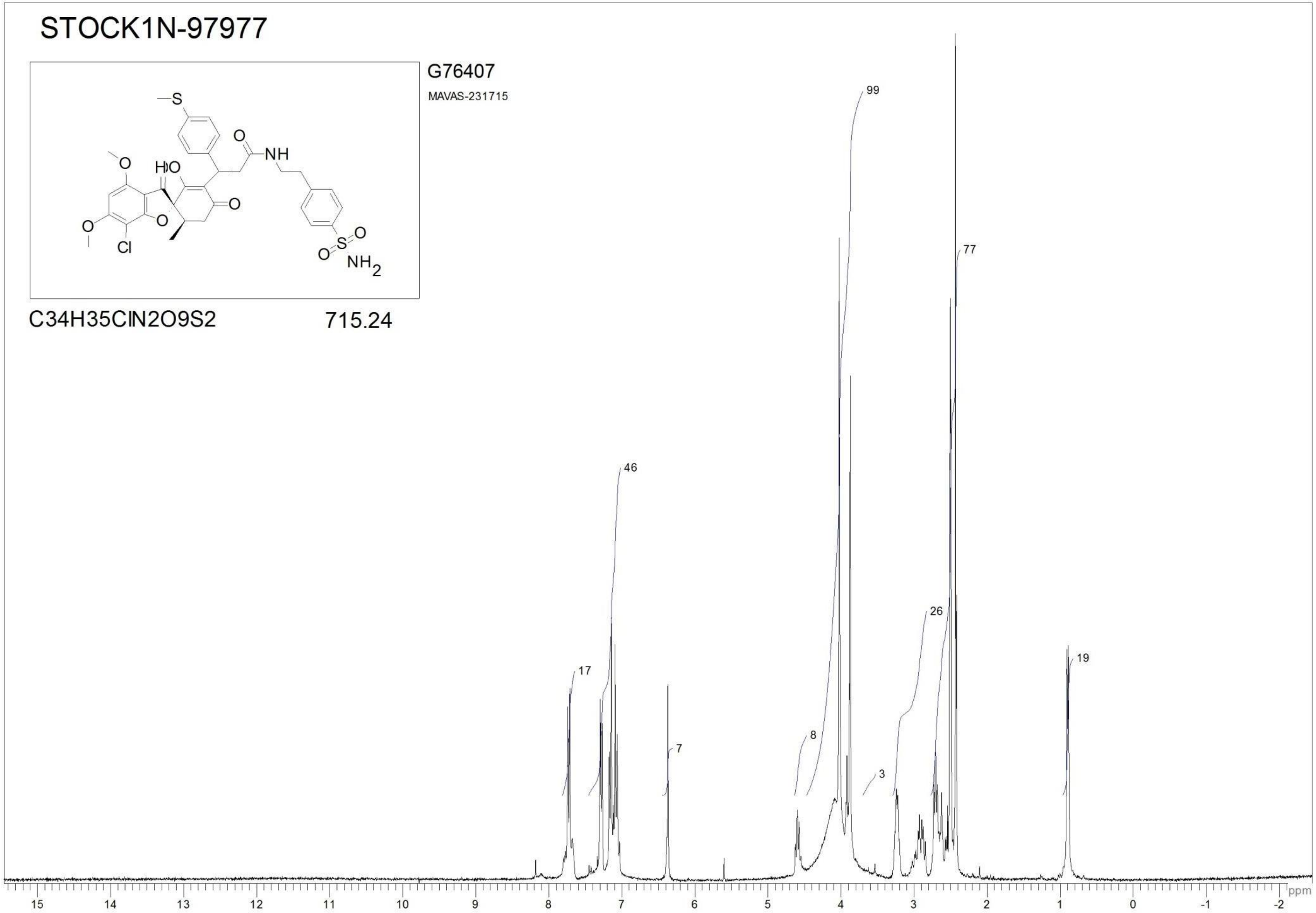

STOCK1N-97977
G76407
MAVAS-231715
C34H35ClN2O9S2
715.24
99
77
46
26
19
17
8
7
3
ppm

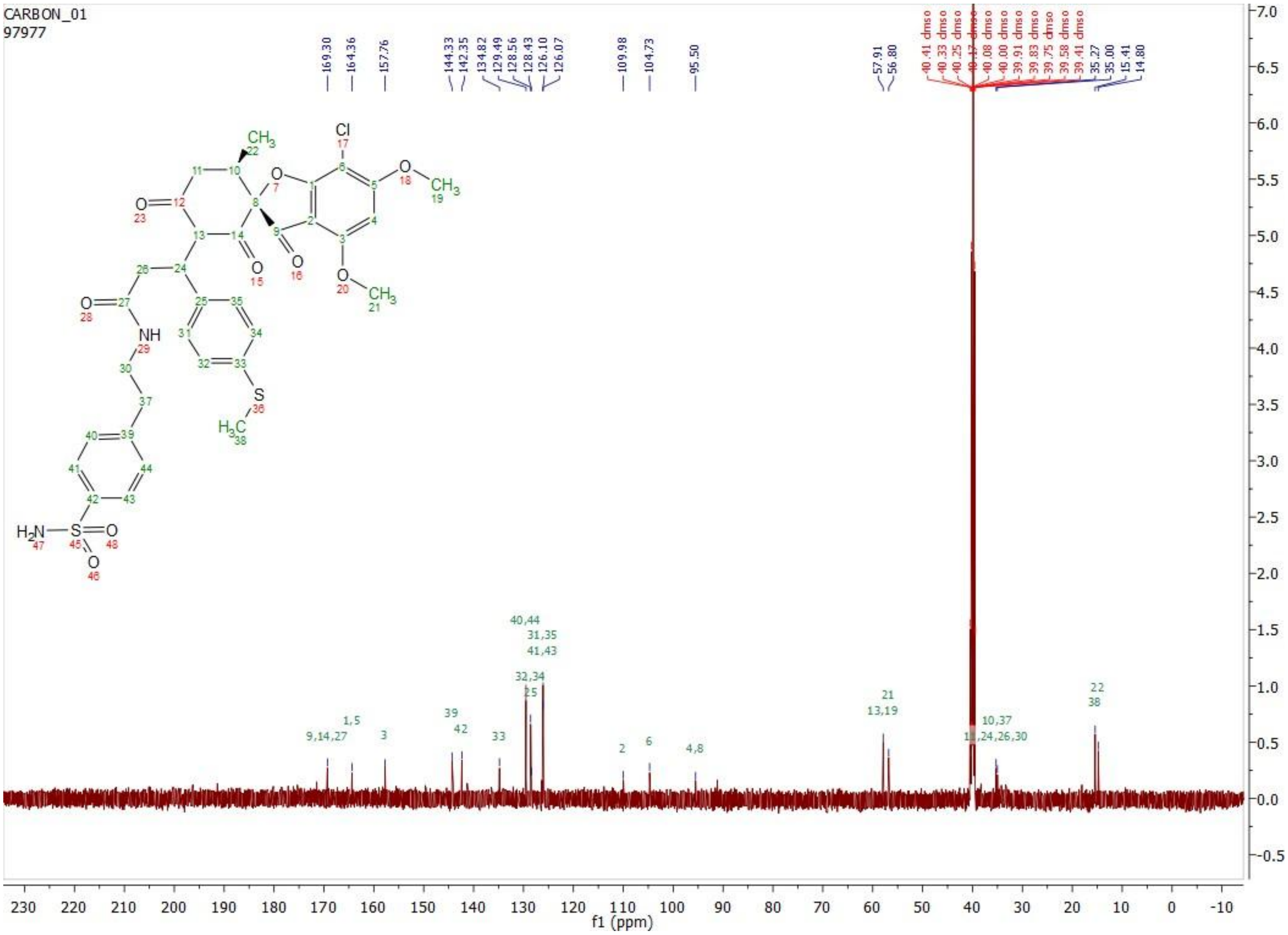
CARBON_01
97977
169.30
164.36
157.76
144.33
142.35
134.82
129.49
128.56
128.43
126.10
126.07
109.98
104.73
95.50
57.91
56.80
40.41 dmso
40.33 dmso
40.25 dmso
40.17 dmso
40.08 dmso
40.00 dmso
39.91 dmso
39.83 dmso
39.75 dmso
39.58 dmso
39.41 dmso
35.27
35.00
15.41
14.80
f1 (ppm)

Compound 1j

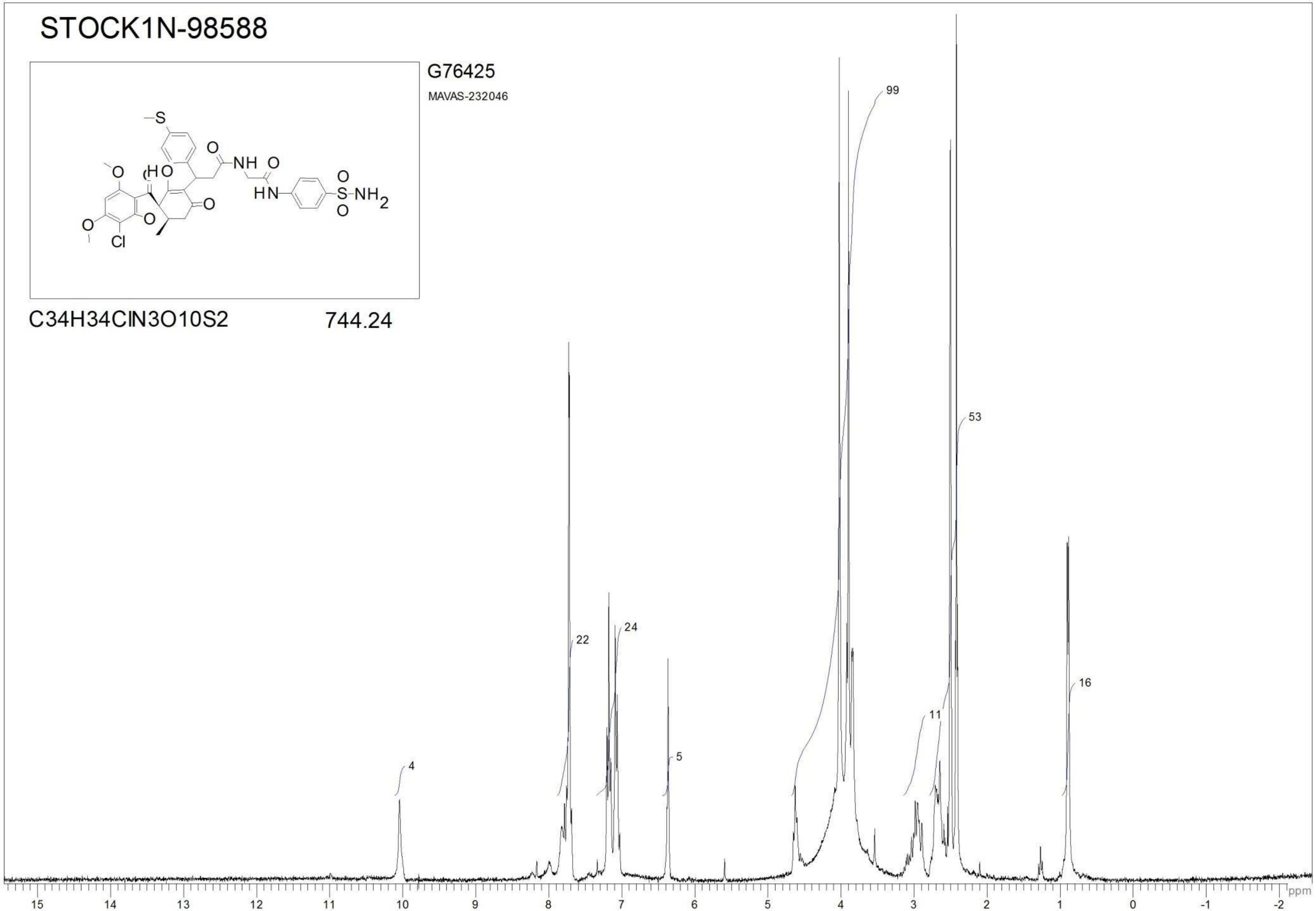

STOCK1N-98588
G76425
MAVAS-232046
C34H34ClN3O10S2
744.24
99
53
22
24
16
11
5
4
ppm
15
14
13
12
11
10
9
8
7
6
5
4
3
2
1
0
-1
-2

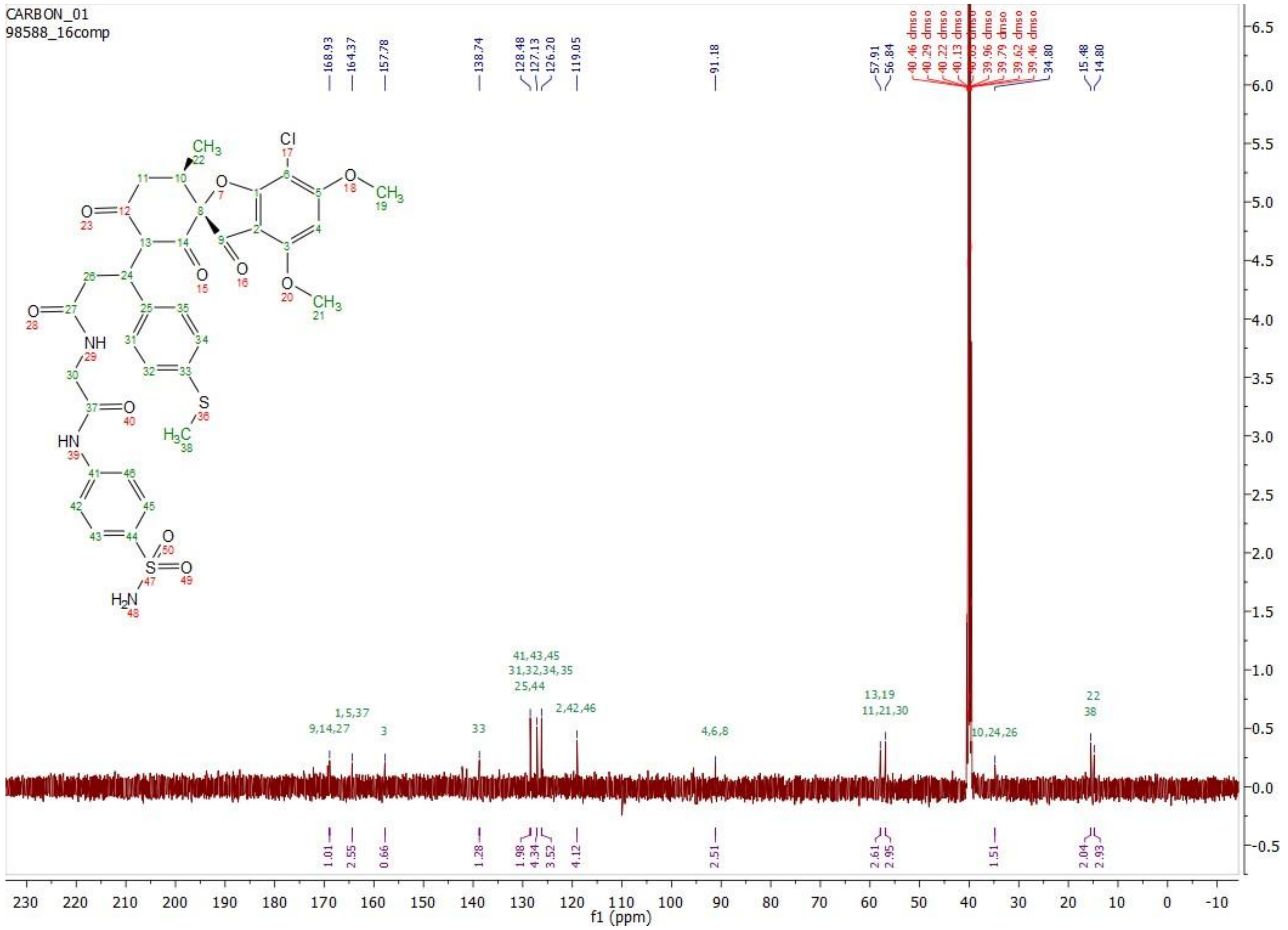

CARBON_01
98588_16comp
168.93
164.37
157.78
138.74
128.48
127.13
126.20
119.05
91.18
57.91
56.84
40.46 dmso
40.29 dmso
40.22 dmso
40.13 dmso
39.96 dmso
39.79 dmso
39.62 dmso
39.46 dmso
34.80
15.48
14.80
41,43,45
31,32,34,35
25,44
2,42,46
1,5,37
9,14,27
3
33
4,6,8
13,19
11,21,30
10,24,26
22
38
1.01
2.55
0.66
1.28
1.98
4.34
3.52
4.12
2.51
2.61
2.95
1.51
2.04
2.93
f1 (ppm)

Compound 4a

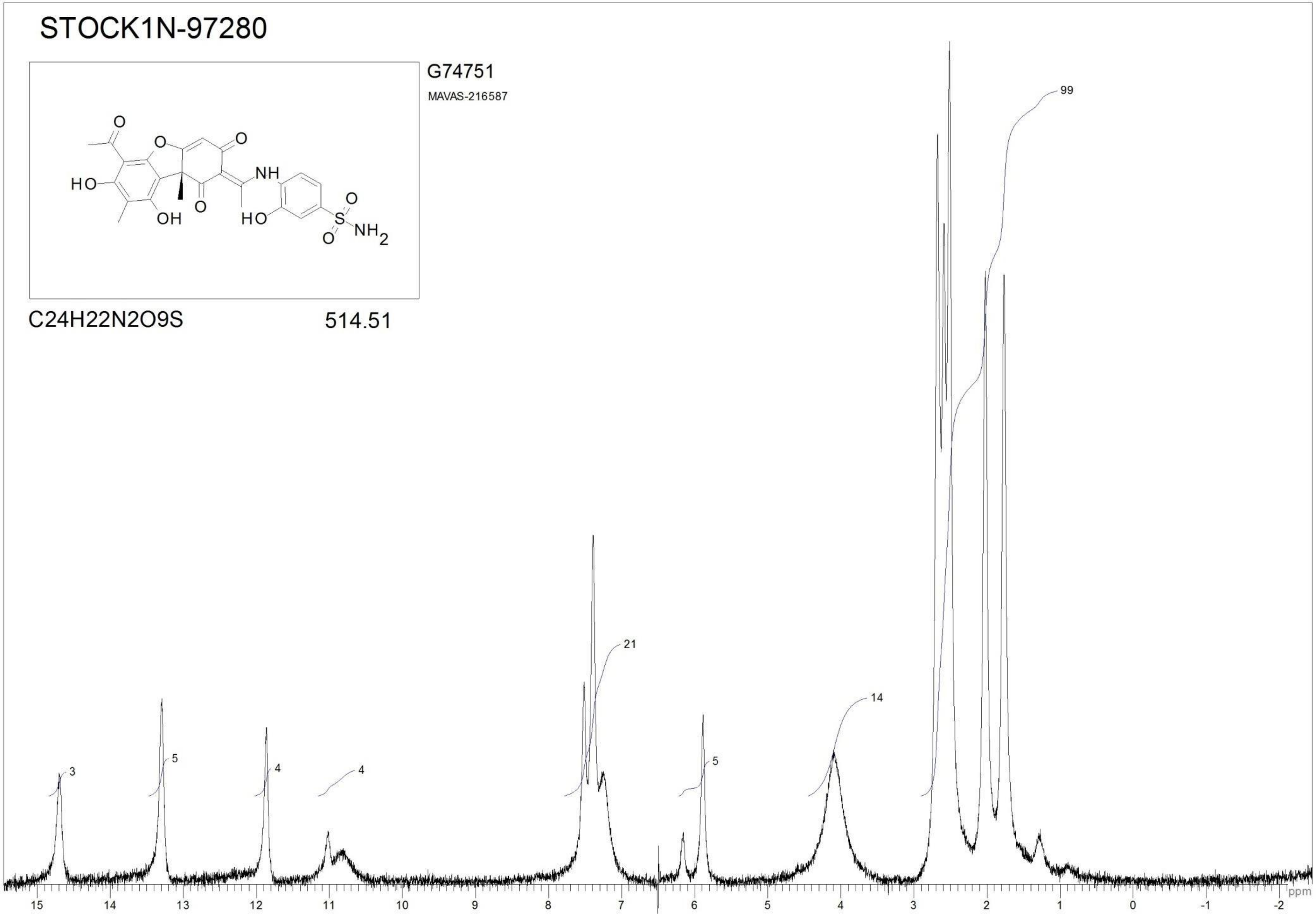

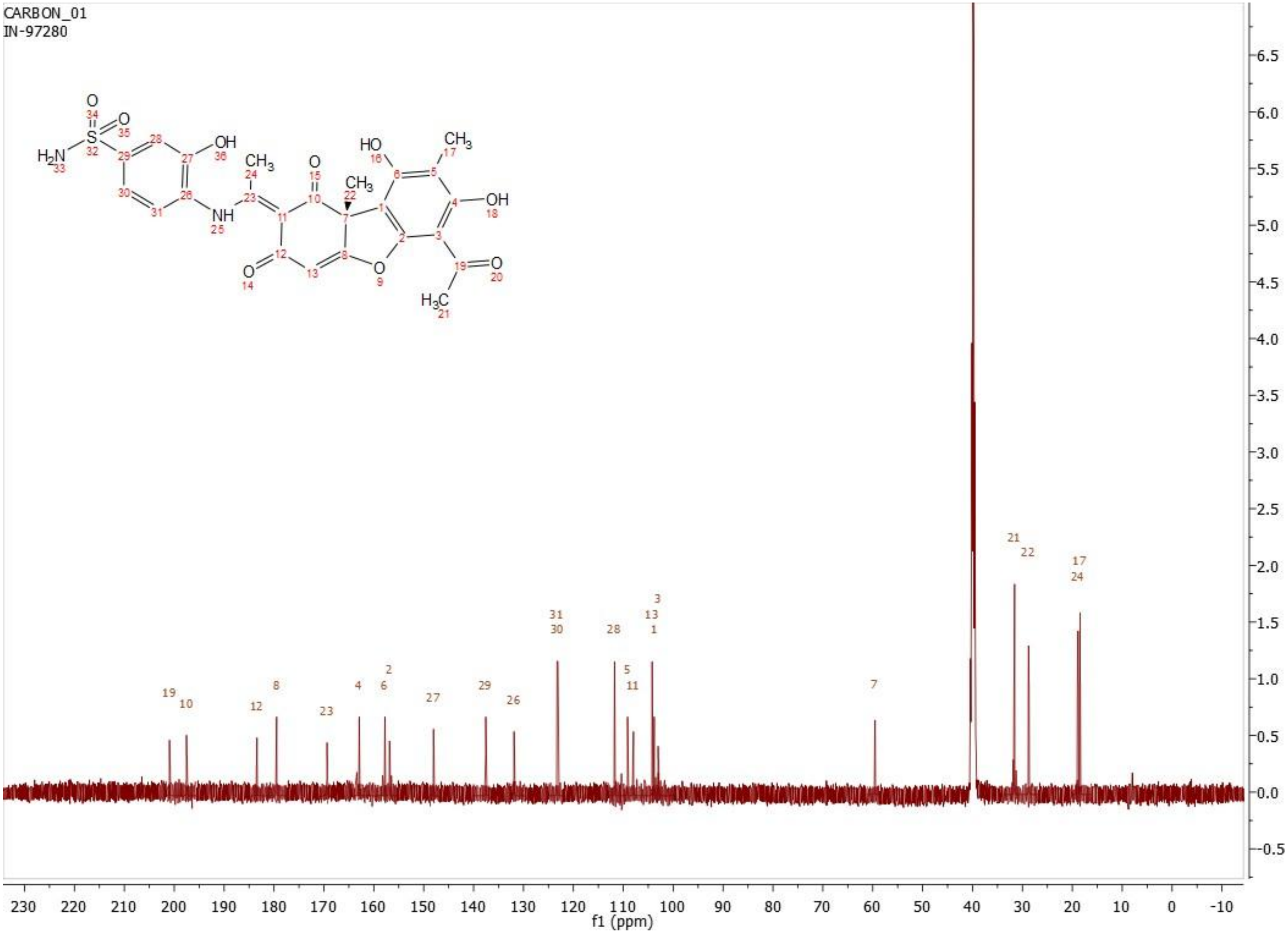
CARBON_01
IN-97280
f1 (ppm)

Compound 4b

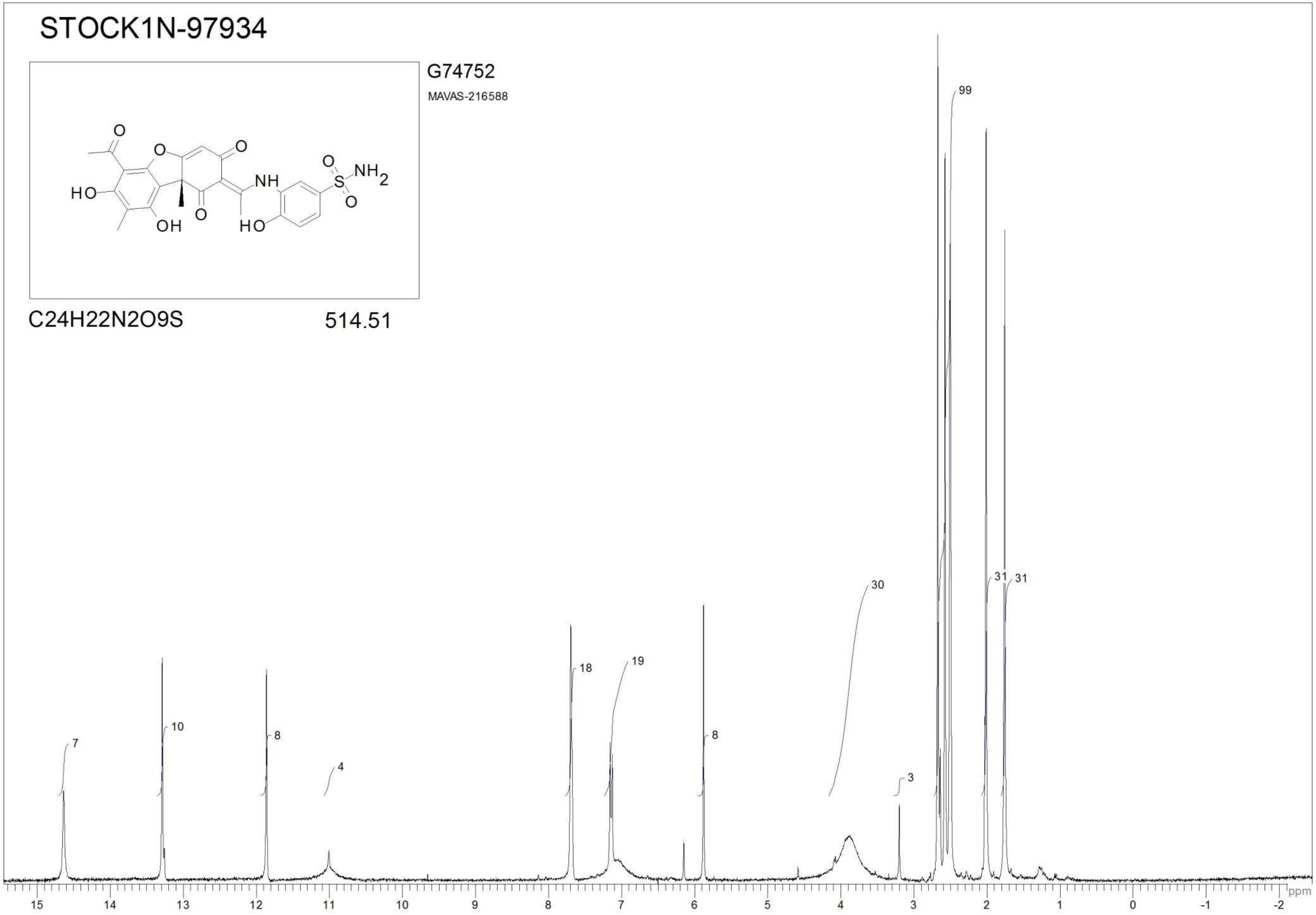
STOCK1N-97934
G74752
MAVAS-216588
C24H22N2O9S
514.51
7
10
8
4
18
19
8
30
3
99
31
31
15
14
13
12
11
10
9
8
7
6
5
4
3
2
1
0
-1
-2
ppm

CARBON_01
97934

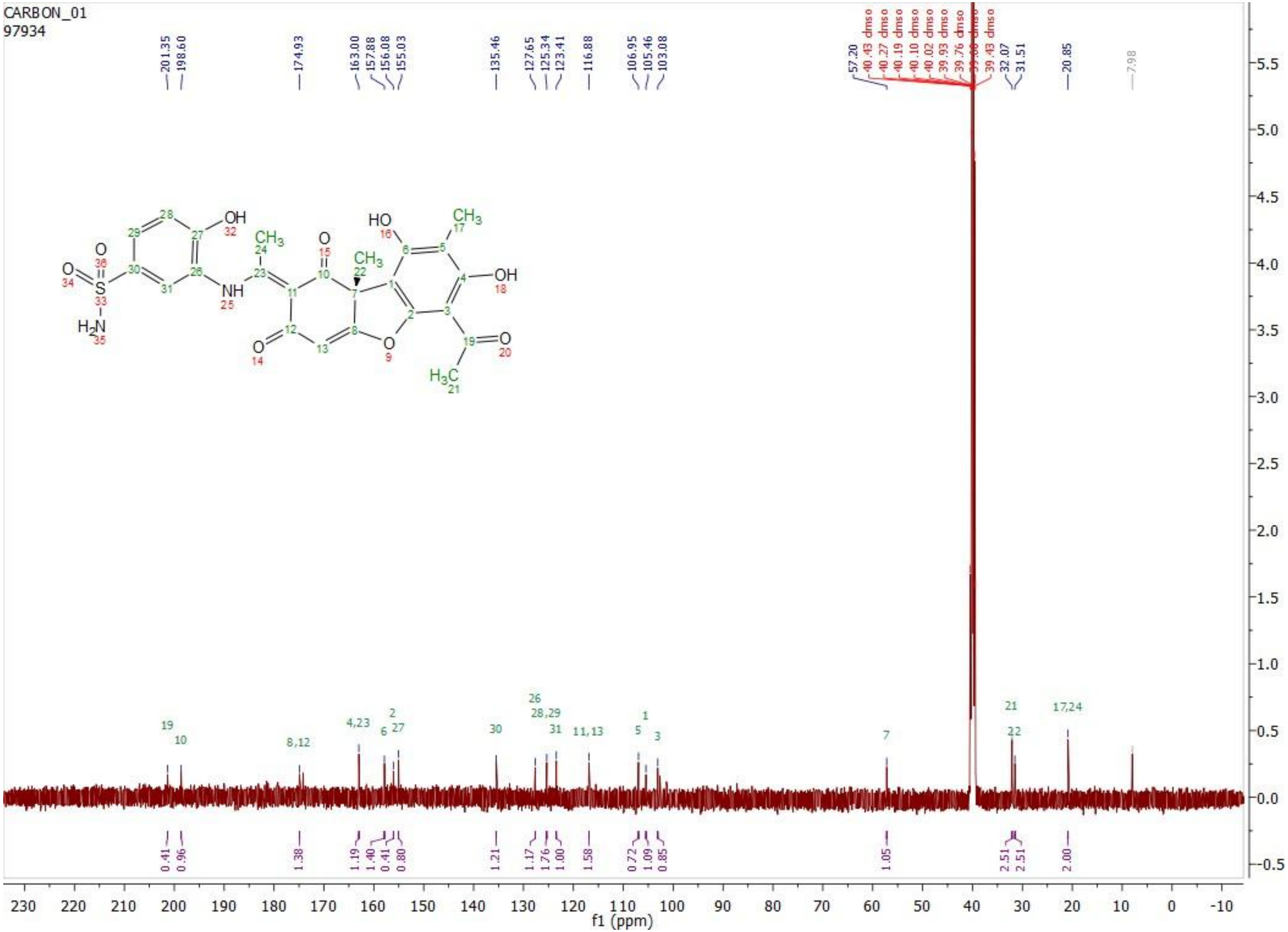

2. Preliminary docking studies

Table S1. Molecular docking free binding energies (kcal/mol) and interactions of designed compounds on hCA I, II and IX isoforms.

Structure	*h*CA isoform	Estimated free binding energy (Kcal/mol)	Structure	*h*CA isoform	Estimated free binding energy (Kcal/mol)
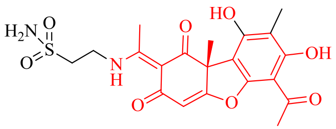	hCA I<br>hCA II<br>hCA IX	-3.20<br>-4.64<br>-3.16	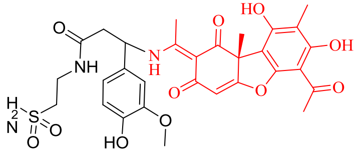	hCA I<br>hCA II<br>hCA IX	-2.67<br>-2.85<br>-4.32
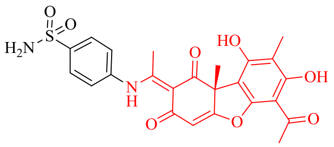	hCA I<br>hCA II<br>hCA IX	-3.71<br>-4.50<br>-3.62	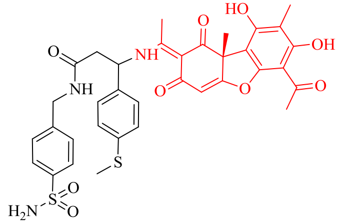	hCA I<br>hCA II<br>hCA IX	-3.60<br>-4.16<br>-5.30
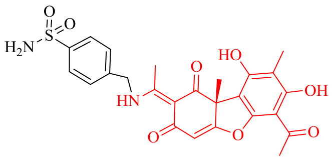	hCA I<br>hCA II<br>hCA IX	-4.88<br>-4.13<br>-5.57	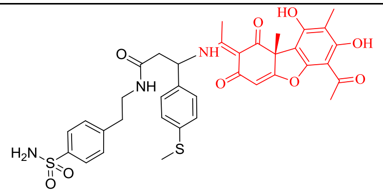	hCA I<br>hCA II<br>hCA IX	-4.95<br>-3.24<br>-3.45
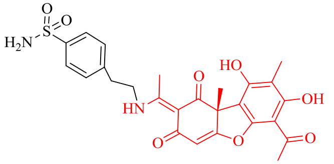	hCA I<br>hCA II<br>hCA IX	-4.29<br>-5.74<br>-5.18	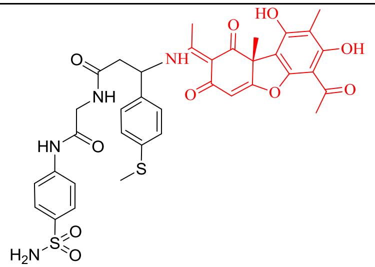	hCA I<br>hCA II<br>hCA IX	-3.87<br>-4.11<br>-5.46
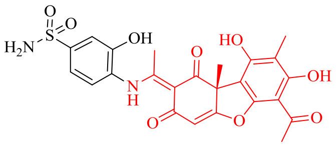<br>1d-4a	hCA I<br>hCA II<br>hCA IX	**-6.72**<br>**-10.23**<br>**-6.57**	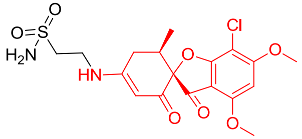	hCA I<br>hCA II<br>hCA IX	**-6.25**<br>**-6.24**<br>**-8.38**
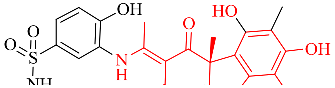	hCA I<br>hCA II	**-5.11**<br>**-4.89**	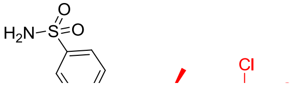	hCA I<br>hCA II	**-5.49**<br>**-7.38**

	hCA IX	**-6.26**		hCA IX	**-6.30**
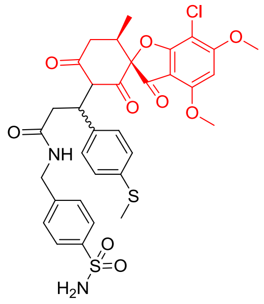	hCA I<br>hCA II<br>hCA IX	**-6.13**<br>**-7.39**<br>**-6.88**	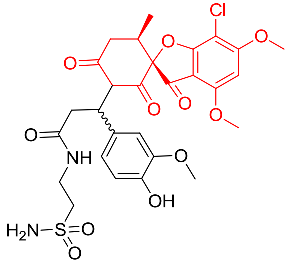	hCA I<br>hCA II<br>hCA IX	**-9.67**<br>**-10.38**<br>**-7.70**
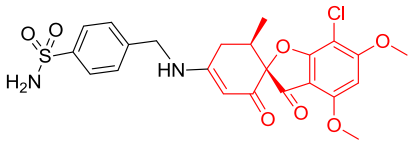	hCA I<br>hCA II<br>hCA IX	**-9.67**<br>**-9.85**<br>**-7.32**	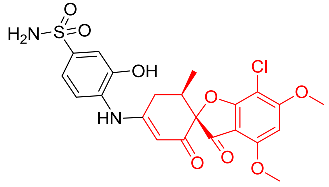	hCA I<br>hCA II<br>hCA IX	**-10.35**<br>**-12.02**<br>**-8.53**
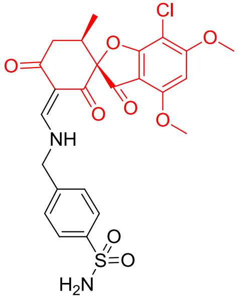	hCA I<br>hCA II<br>hCA IX	**-6.28**<br>**-5.89**<br>**-8.52**	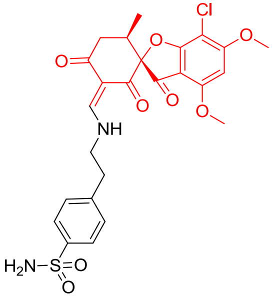	hCA I<br>hCA II<br>hCA IX	**-5.76**<br>**-6.78**<br>**-8.36**
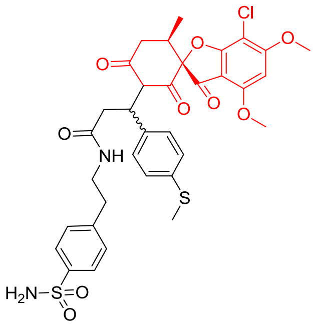	hCA I<br>hCA II<br>hCA IX	**-12.45**<br>**-11.64**<br>**-12.74**	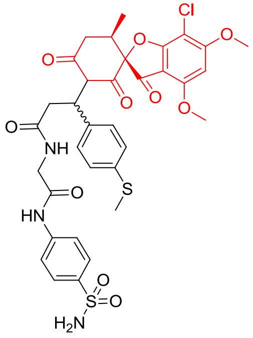	hCA I<br>hCA II<br>hCA IX	**-8.28**<br>**-12.87**<br>**-12.02**
**AAZ**	hCA I	-8.28			

	hCA II	-8.87
	hCA IX	-9.02

3. Prediction of toxicity

Table S2. Prediction of toxicity.

No	Predicted LD50 (mg/kg)	Predicted Toxicity Class	Hepatotoxicity	Carcinogenicity	Mutagenicity	Cytotoxicity
**1a**	1100	IV	Inactive (0.55)	Inactive (0.58)	Inactive (0.57)	Inactive (0.60)
**1b**	2000	IV	Inactive (0.54)	Inactive (0.61)	Inactive (0.67)	Inactive (0.74)
**1c**	1100	IV	Inactive (0.53)	Inactive (0.62)	Inactive (0.64)	Inactive (0.66)
**1d**	2000	IV	Inactive (0.54)	Inactive (0.60)	Inactive (0.68)	Inactive (0.73)
**1e**	3500	V	Inactive (0.55)	Inactive (0.59)	Inactive (0.67)	Inactive (0.64)
**1f**	3500	V	Inactive (0.55)	Inactive (0.61)	Inactive (0.66)	Inactive (0.65)
**1g**	1100	IV	Inactive (0.55)	Inactive (0.60)	Inactive (0.63)	Inactive (0.60)
**1h**	5000	V	Inactive (0.52)	Inactive (0.62)	Inactive (0.69)	Inactive (0.64)
**1i**	5000	V	Inactive (0.53)	Inactive (0.63)	Inactive (0.68)	Inactive (0.64)
**1j**	5000	V	Inactive (0.56)	Inactive (0.63)	Inactive (0.68)	Inactive (0.64)
**4a**	838	IV	Inactive (0.54)	Inactive (0.58)	Inactive (0.67)	Inactive (0.74)
**4b**	838	IV	Inactive (0.54)	Inactive (0.58)	Inactive (0.67)	Inactive (0.74)

Number in brackets indicate possibilities.

4. Molecular Docking Studies

Software Used

The ligand preparations done by using chemdraw 12.0, geometries were optimized using LigandScout 4.4.5. The "Build/check/repair model" to the session "Prepare PDB file for docking programs" module was used for proteins preparation. For the finally preparation of both ligands and protein preparation Wizard of AutoDock tools 1.5.6 are used. Autodock 4 (ver. 4.2.6) was employed for docking simulations and Autogrid4 for affinity grid maps preparation [1]. The resulting poses and potential interactions were visualized using the LigandScout 4.4.5 program.

Protein and ligand preparation

X-ray crystal structures of *h*CA I (PDB code 3W6H) and *h*CA II (PDB code 3HS4) cytosolic isoforms as well as *h*CA IX (PDB code 3IAI) transmembrane tumor-associated isoform were retrieved from Brookhaven Protein Data Bank (PDB) [2]. The pdb files of proteins were submitted to "Build/check/repair model" to the session "Prepare PDB file for docking programs" and missing side chains were modeled in, water positions and symmetry were corrected, and hydrogen atoms were added. Only chain A of each enzyme of the repaired pdb file was evaluated and passed to AutodockTools (ADT ver.1.5.6) for pdbqt file preparation. ADT assigned polar hydrogens, water molecules and non-standard residues were removed, only polar hydrogen was maintained, and Gasteiger charges were computed for protein atoms. AutoDock saved the prepared file in PDBQT format.

All the molecules were sketched in chemdraw 12.0 program. The geometry of built compounds was optimized using the molecular mechanical force fields 94 (MMFF94) energy via program LigandScout, partial charges were also calculated, comformers of each ligand were generated and the one with the best conformation was maintained and saved as mol2 files that were passed, as usual, to ADT for pdbqt file preparation. There, polar hydrogen was added to each structure, followed by computing Gasteiger and Kollman charges, and the torsions.

Docking Procedure

Autodock4 (ver. 4.2.6) was employed for docking simulations. A computationally (relatively) 'hybrid' force field that contains terms based on molecular, mechanics and empirical terms is used by AutoDock. The evaluation step in a nutshell includes: first, calculation of the energy of protein and ligand in the unbound state. Second, calculation of the energy of the ligand–protein complex and take the difference between first and second step.

$$\Delta G = (V_{bound}^{L-L} - V_{unbound}^{L-L}) + (V_{bound}^{P-P} - V_{unbound}^{P-P}) + (V_{bound}^{P-L} - V_{unbound}^{P-L} + \Delta S_{conf})$$

where P refers to the protein, L refers to the ligand, V are the pair-wise evaluations mentioned above and ΔS_{conf} denotes the loss of conformational entropy upon binding [3]. The ligand molecule is in an arbitrary conformation, orientation and position and this molecular docking program finds favorable poses in a protein-binding site using Lamarckian genetic algorithms implemented therein to search for the best conformers.

Lamarckian genetic algorithm was used as search engine, with a total of 100 runs. The region of interest, used by Autodock4 for docking runs and by Autogrid4 for affinity grid maps preparation, was defined in such a way to comprise the whole catalytic binding site using a grid of 50 x 50 x 50 points with a grid space of 0.375 Å. All parameters used in docking were default. The translation, quaternion and torsions steps were taken from default values in AutoDock. The Lamarckian genetic algorithm and the pseudo-Solis and Wets methods were applied for minimization using default parameters. The number of docking runs was 100. After docking, the 100 solutions were clustered into groups with RMS lower than 1.0 E. The clusters were ranked by the lowest energy representative of each cluster. Upon completion of docking, the best poses were screened by examination of binding energy ($\Delta G_{binding}$, kcal/mol) and number in cluster. In order to describe the ligand-binding pocket interactions, the top ranked binding mode found by AutoDock in complex with the binding pocket of enzyme was selected. The resulting poses and potential interactions were visualized using the LigandScout 4.4.5. Finally, the docking protocol was verified by re-docking of the co-crystallized ligand acetazolamide (AAZ) in the vicinity of the active sites of each enzyme with RMSD values 0.885, 0.966 and 1.034 Å for hCA I, II and IX, respectively.

1. Morris, G. M., Huey, R., Lindstrom, W., Sanner, M. F., Belew, R. K., Goodsell, D. S. and Olson, A. J. (2009) Autodock4 and AutoDockTools4: automated docking with selective receptor flexiblity. *J. Computational Chemistry* **2009**,*16*: 2785-91.
2. http://www.rcsb.org/. (Accessed 19/6/2021).
3. R. Huey, G.M. Morris, A.J. Olson, and D.S. Goodsell, A semiempirical free energy force field with charge-based desolvation, J. Comp. Chem. 28 (2007), pp. 1145–1152.
